# RNA modification in normal hematopoiesis and hematologic malignancies

**DOI:** 10.1002/mco2.787

**Published:** 2024-10-23

**Authors:** Xi Chen, Yixiao Yuan, Fan Zhou, Lihua Li, Jun Pu, Xiulin Jiang

**Affiliations:** ^1^ Department of Neurosurgery The Second Affiliated Hospital of Kunming Medical University Kunming China; ^2^ NHC Key Laboratory of Drug Addiction Medicine Kunming Medical University Kunming Yunnan China; ^3^ Department of Medicine UF Health Cancer Center University of Florida Gainesville Florida USA; ^4^ Department of Medicine and Department of Biochemistry and Molecular Biology University of Florida Gainesville Florida USA

**Keywords:** hematopoietic stem cells, leukemia stem cells, potential targets, RNA modification, signaling pathway

## Abstract

N6‐methyladenosine (m6A) is the most abundant RNA modification in eukaryotic cells. Previous studies have shown that m6A plays a critical role under both normal physiological and pathological conditions. Hematopoiesis and differentiation are highly regulated processes, and recent studies on m6A mRNA methylation have revealed how this modification controls cell fate in both normal and malignant hematopoietic states. However, despite these insights, a comprehensive understanding of its complex roles between normal hematopoietic development and malignant hematopoietic diseases remains elusive. This review first provides an overview of the components and biological functions of m6A modification regulators. Additionally, it highlights the origin, differentiation process, biological characteristics, and regulatory mechanisms of hematopoietic stem cells, as well as the features, immune properties, and self‐renewal pathways of leukemia stem cells. Last, the article systematically reviews the latest research advancements on the roles and mechanisms of m6A regulatory factors in normal hematopoiesis and related malignant diseases. More importantly, this review explores how targeting m6A regulators and various signaling pathways could effectively intervene in the development of leukemia, providing new insights and potential therapeutic targets. Targeting m6A modification may hold promise for achieving more precise and effective leukemia treatments.

## Graphical abstract

N6‐methyladenosine (m6A) is the most abundant RNA modification in eukaryotic cells. Previous studies have shown that m6A plays a critical role under both normal physiological and pathological conditions. This review systematically reviews the latest research advancements on the roles and mechanisms of m6A regulatory factors in normal hematopoiesis and related malignant diseases. Targeting m6A modification may hold promise for achieving more precise and effective leukemia treatments.

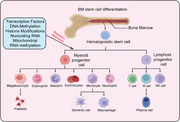

## 1 INTRODUCTION

Chemical are highly specific and effective methods for regulating the functions of biological macromolecules.^1^ All biological macromolecules, including proteins, RNA, DNA, sugars, and lipids, are influenced by postsynthetic covalent modifications.^2^ Recent studies have shown that RNA modifications plays a crucial role not only as an intermediary in protein synthesis but also in posttranscriptional regulation, directly affecting gene expression.^3^ RNA methylation is a significant form of RNA modification, encompassing N6‐methyladenosine (m6A), N1‐methyladenosine (m1A), 5‐methylcytidine (m5C), pseudouridine (Ψ), N7‐methylguanosine (m7G), and 5‐hydroxymethylcytosine (hm5C), all of which have been reported to impact various biological processes.^4^, ^5^, ^6^, ^7^, ^8^, ^9^ The m6A modification is one of the most thoroughly studied among these modifications in recent years and plays a crucial role in disease progression.

m6A RNA modification represents a novel mechanism for regulating gene expression in eukaryotes. m6A modification was first identified in the 1970s^10^ and is a prevalent modification in both messenger RNA (mRNA) and noncoding RNA (ncRNA). Nearly one‐third of mammalian mRNA contains m6A modifications, with an average of 3–5 m6A sites per mRNA. Many of these m6A sites are highly conserved between mice and humans. The distribution of m6A modifications across the transcriptome is not random; m6A sites feature the characteristic DRACH sequence motif (D = G, A, or U; R = G or A; H = A, C, or U) and are primarily enriched in coding regions and the untranslated regions at the 3′ end (3′UTR).^11^, ^12^ m6A modification is a dynamic and reversible process, governed by methyltransferases (writers) and demethylases (erasers), which respectively add and remove methyl groups from RNA. These modifications are recognized by specific binding proteins (readers). As the most abundant RNA modification, m6A regulates various aspects of mRNA metabolism, including mRNA degradation, transport, and translation.^13^, ^14^, ^15^, ^16^ Through distinct mechanisms, m6A modifications modulate downstream signaling pathways, activate target gene expression, and ultimately participate in normal physiological processes or pathological conditions. Therefore, understanding the complex regulatory mechanisms of m6A modification is critical for elucidating its role in the transition between normal physiological and abnormal pathological states.

The hematopoietic system is one of the most vital systems for maintaining life activities, providing oxygen and nutrients to the body, maintaining internal homeostasis through material exchange, and offering immune defense and protection.^17^ Blood cells, a crucial component of blood, can be divided into myeloid and lymphoid lineages.^18^ Myeloid cells include erythroid cells, megakaryocytes, granulocytes, and monocytes, while lymphoid cells primarily consist of T lymphocytes, B lymphocytes, and natural killer (NK) cells.^19^ All mature blood cells originate from hematopoietic stem cells (HSCs), a group of multipotent stem cells with self‐renewal and differentiation potential. The process of production, development, and maturation of HSCs and various blood cells is termed hematopoiesis, which begins in early embryonic development and continues throughout life.^20^ Any disruption at any stage can lead to hematological diseases. Currently, for malignant hematological diseases like leukemia, HSC transplantation is the most common treatment, with its efficacy largely depending on the quality and quantity of the transplanted cells.^21^ Thus, inducing the production of HSCs in vitro and maintaining their stemness becomes an essential solution. Studying the developmental processes and molecular regulatory mechanisms of HSCs in vivo can provide theoretical support for obtaining a large number of transplantable HSCs in vitro.

Acute myeloid leukemia (AML) is the most common and deadly form of acute leukemia in adults, characterized by the abnormal clonal proliferation of primitive HSCs/downstream progenitor cells, which is a critical mechanism of its development and progression and a recent research focus.^22^ Clinically, AML treatment still primarily relies on traditional chemotherapy, supplemented by immunotherapy or allogeneic stem cell transplantation.^23^ Over the past few decades, although the initial complete remission rate and overall survival rate of AML have improved, the side effects of chemotherapy, postchemotherapy body conditions, quality of life, and high recurrence rates remain significant challenges in modern medicine.^24^ Leukemia stem cells (LSCs), identified through their stem cell properties such as drug resistance, self‐renewal, and undifferentiated state, were first successfully isolated in 1994.^25^ These cells possess self‐renewal capability, robust differentiation potential, and the ability to generate heterogeneous leukemia cell populations.^26^ Recent studies have found that LSCs play a critical role in the development, progression, and relapse of AML, potentially persisting after chemotherapy and driving clonal diversity, leading to more aggressive disease forms and fatal outcomes.^27^ It is currently believed that the persistent existence of LSCs is one of the primary causes of relapse and resistance in leukemia. Most LSCs remain in a quiescent state, rendering them highly resistant to conventional chemoradiotherapy. These cells play a crucial role in the initiation, progression, and recurrence of leukemia, leading to treatment failure and contributing to the high mortality rate associated with the disease. The m6A modification plays a crucial role in the self‐renewal of LSCs and the progression of AML. Therefore, targeting m6A methylation and LSCs to impair their function is a promising and meaningful research direction with significant practical implications.

Although RNA m6A methylation modification has increasingly been recognized as crucial in the regulation of gene expression, its role in controlling cell fate decisions under various normal physiological and pathological conditions remains unclear. With the rapid advancement of high‐throughput m6A sequencing technologies in recent years, accumulating evidence suggests a strong association between m6A modification regulation and the self‐renewal of HSCs, as well as the development and progression of hematologic malignancies. Therefore, in this review, we systematically summarize these new findings and comprehensively outline the research progress on the role of RNA m6A methylation modification in normal hematopoiesis and hematologic malignancies. We further discuss the potential significance of targeting m6A modification regulators in the treatment of leukemia, providing a scientific basis for developing novel molecular‐targeted therapies aimed at abnormal m6A modifications in related hematologic tumors.

## 2 RNA MODIFICATION

RNA modifications refer to chemical modifications occurring on RNA molecules, which significantly influence the stability, localization, translation, and overall function of RNA. Common RNA modifications include m6A, m1A, m5C,Ψ, m7G, and hm5C, all of which have been reported to impact various biological processes. These modifications play critical roles in the posttranscriptional regulation of gene expression, contributing to processes such as cell differentiation, development, and stress responses. Dysregulation of RNA modifications has been implicated in a wide range of diseases, including cancer, neurological disorders, and metabolic diseases. As research progresses, RNA modifications are increasingly recognized as potential therapeutic targets for disease treatment and as biomarkers for diagnosis. Here, we systematically review the composition and regulatory factors of common RNA chemical modifications, aiming to provide insights into these modifications and offer theoretical guidance for understanding disease mechanisms.

### 2.1 RNA m6A modification

**FIGURE 1:**
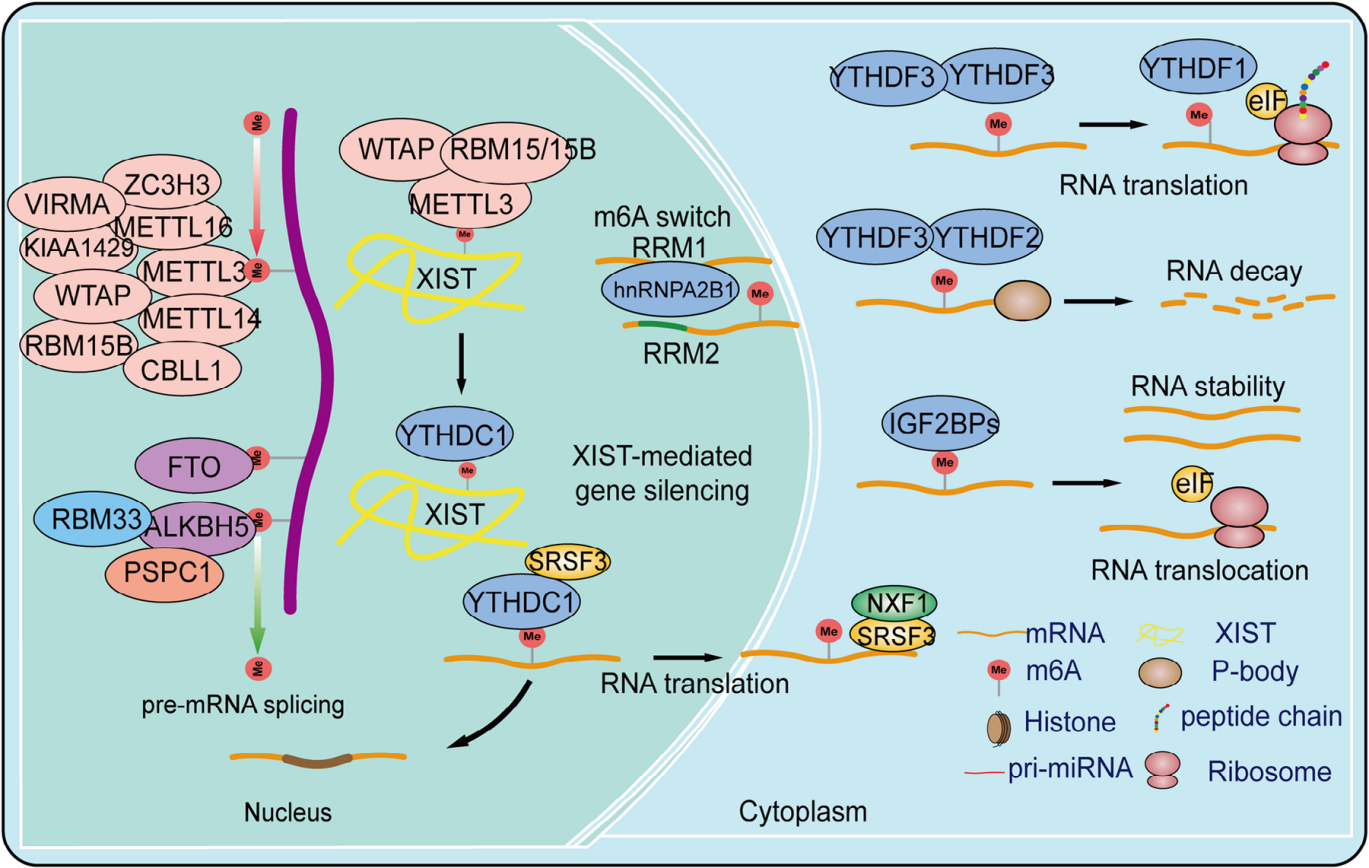
The composition of the m6A modification machinery, including m6A methyltransferases, m6A demethylases, and m6A reader proteins, collectively forming the regulatory network that governs RNA metabolism. METTL3, methyltransferase‐like3; METTL14, methyltransferase‐like14; WTAP, Wilms tumor1‐associating protein; METTL5, methyltransferase‐like 5; METTL16, methyltransferase‐like 16; ZCCHC4, zinc finger CCHC domain‐containing protein4; RBM15, RNA binding motif protein15; FTO, fat mass and obesity‐associated protein; ALKBH5, human AlkB homolog 5; PRMT5, protein arginine methyltransferase 5; YTHDF1, YT521‐B homology (YTH) domain‐containing family protein 1; YTHDF2, YT521‐B homology (YTH) domain‐containing family protein 2; YTHDF3, YT521‐B homology (YTH) domain‐containing family protein 3;YTHDC1, YT512‐B homology domain‐containing protein 1; IGF2BPs, insulin‐like growth factor 2 mRNA‐binding protein; HNRNPA2B1, heterogeneous nuclear ribonucleoprotein A2B1; NXF1, nuclear RNA export factor 1; SRSF3, serine/arginine‐rich splicing factor 3; RRM2, ribonucleotide reductase regulatory subunit M2; XIST, X‐inactive specific transcript; M6A, N6‐methyladenosine; RBM33, RNA binding motif protein 33; PSPC1, paraspeckle component 1.

m6A modification is one of the most crucial and widespread types of RNA epigenetic regulation, primarily occurring at the N6 position of adenine in mRNA. By influencing RNA stability, splicing, nuclear export, and translation efficiency, m6A modification plays a significant role in regulating gene expression and cell fate determination. This regulation at the RNA level provides new insights into the complexity and diversity of gene expression. Further investigation into the specific mechanisms and functions of m6A modification holds promise for the development of novel therapeutic strategies, particularly in cancer treatment, where targeting m6A modification and its regulatory factors offers a broad prospect for precision medicine. Here, we systematically summarize the composition of m6A modifications, their regulatory factors, and their biological functions (Figure 1).

#### 2.1.1 m6A methyltransferases

m6A writers are a class of methyltransferases that catalyze the formation of m6A by assembling m6A onto target RNA through a complex. The m6A methyltransferase complex (MTC) consists of a heterodimer of methyltransferase‐like protein 3 (METTL3) and METTL14, Wilms tumor 1‐associated protein (WTAP), RNA binding motif protein 15 (RBM15), RBM15B, Cbl proto‐oncogene like 1 (CBLL1, also known as HAKAI), Vir‐like m6A methyltransferase associated (VIRMA, also known as KIAA1429), and zinc finger CCCH‐type containing 13 (ZC3H13).^2^, ^28^, ^29^ METTL3 serves as the catalytic core of the m6A MTC, using S‐adenosyl methionine (SAM) as the methyl donor. METTL14 provides structural support for METTL3 and forms a stable heterodimer core complex, which is crucial for substrate recognition.^30^ The METTL3–METTL14 dimer mediates the deposition of m6A on mammalian RNA, functioning as both oncogenes and tumor suppressors in different tumors or even within the same tumor. Cytoplasmic METTL3 directly promotes the translation of several oncogenes, including epidermal growth factor receptor (EGFR) and Hippo pathway effector TAZ.^31^ In AML, high METTL3 expression plays a critical role in AML cell proliferation, maintaining the undifferentiated phenotype of AML cells, and is essential for AML development in mouse xenograft models.^32^ At the molecular level, METTL3 promotes translation of oncogenic target genes, such as modifying the mRNA encoding the oncogenic transcription factor MYC.^33^ In immunodeficient mice, downregulation of METTL3 leads to cell cycle arrest, leukemia cell differentiation, and an inability to establish a leukemia model. Although METTL14 is not significantly expressed in leukemia, it also acts as an oncogene in AML, functioning in a MYC‐dependent manner.^34^, ^35^ In liver cancer, METTL3 overexpression drives tumor growth by promoting the degradation of SOCS2 mRNA and upregulating SNAIL translation, leading to epithelial–mesenchymal transition (EMT) in cancer cells.^36^ In contrast, low expression of METTL14 has been shown to have the opposite effect, reducing the metastatic potential of liver cancer cells by interacting with the microprocessor complex DGCR8 and promoting miRNA maturation ZC3H13.^37^ METTL3 oncogenic role has also been reported in lung cancer, where it is overexpressed in human lung cancer tissues compared with normal tissues, and METTL14 protein and transcript abundance are also increased.^38^, ^39^

WTAP, lacking methylation activity, acts as a regulatory subunit of the complex, recruiting the METTL3–METTL14 complex to form the catalytic core targeting RNA. Knockout and overexpression of METTL3 both lead to upregulation of WTAP protein expression, indicating that METTL3 levels play a critical role in WTAP protein homeostasis.^40^ However, merely upregulating WTAP without functional METTL3 is insufficient to promote cell proliferation, suggesting that WTAP oncogenic function is closely related to the functional m6A methylation complex. WTAP influences MAPK, AKT, Wnt, and nuclear factor kappa‐B (NF‐κB) signaling pathways, promoting tumor progression by regulating downstream targets such as EGR3, HK2, ETS1, and CAV‐1.^41^ RBM15, a member of the SPEN family located at chromosome 1p13.3, encodes homologs of the RNA‐binding protein RBM15/RBM15B.^42^ RBM15/RBM15B, interacting partners of WTAP, recruit the complex to specific RNA sites by binding to U‐rich RNA consensus sequences, mediating m6A formation in XIST and cellular mRNA.^42^ Knockdown of RBM15/15B significantly reduces overall m6A levels, indicating their crucial role in the MTC. In tumor progression, the role of RBM15 in the MTC has been reported only in leukemia, liver cancer, and laryngeal cancer.^43^, ^44^ KIAA1429 (VIRMA) is a key subunit of the MTC, functioning as a scaffold by recruiting the MTC to the 3′ UTR and near the stop codon. KIAA1429 selectively mediates methylation and promotes liver cancer progression and metastasis by inducing m6A methylation on the 3′ UTR of GATA3 precursor mRNA, leading to its degradation.^45^ Additionally, KIAA1429 is significantly upregulated in liver cancer tissues and inhibits ID2 expression by increasing m6A levels in ID2 mRNA, thereby promoting liver cancer cell migration and invasion.^46^ Recent studies have shown that ZC3H13, a transcription factor with a conserved zinc finger structure, plays a critical role in RNA m6A methylation by mediating the nuclear localization of the ZC3H13‐WTAP complex.^47^ This factor is highly expressed in various cancers and is associated with prognosis. ZC3H13 mainly promotes the binding of the MTC to RNA and has been shown to have tumor suppressor effects, inhibiting colorectal and breast cancer progression and metastasis through regulation of Ras–ERK and Wnt signaling pathways.^48^, ^49^ Furthermore, METTL16 has been identified as the methyltransferase for U6 small nuclear RNA (snRNA), regulating SAM homeostasis by catalyzing substrates with the “UACAGAGAA” sequence. Under conditions of SAM deficiency, METTL16 induces splicing of a retained intron, promoting MAT2A expression and increasing SAM levels. However, the role of METTL16 in cancer requires further investigation.^50^ In summary, the m6A modification introduced by the m6A MTC plays a crucial role in regulating RNA metabolism, including RNA splicing, stability, transport, and translation efficiency. Dysregulation of m6A methylation has been closely associated with various diseases.

#### 2.1.2 m6A demethylases

In 2011, Wei et al.^51^ first demonstrated in vivo that the fat mass and obesity‐associated protein (FTO) can reverse m6A, making FTO the first identified m6A demethylase. In 2017, FTO's role in tumor progression was first reported. Studies have shown that FTO reduces m6A levels on ASB2 and RARA mRNA transcripts, regulating their expression and promoting AML progression.^52^ Additionally, FTO promotes tumor progression in liver, lung, breast, cervical, and colorectal cancers (CRCs) while acting as a tumor suppressor in renal, pancreatic, thyroid, and cholangiocarcinomas.^43^ Following FTO, ALKBH5 was identified as the second m6A demethylase, involved in the biological progression of various cancers and exhibiting both oncogenic and tumor‐suppressive functions.^53^ In breast cancer, ALKBH5 expression is induced by hypoxia‐inducible factor 1α (HIF1α) and HIF1β, and its overexpression under hypoxic conditions reduces NANOG mRNA methylation levels, increasing the number of breast cancer stem cells.^53^ RBM33, a recently identified RNA‐binding protein associated with substrate selectivity for ALKBH5, can act as an m6A recognition protein, binding to m6A‐modified RNA substrates and recruiting ALKBH5 for demethylation. Moreover, SUMOylation significantly inhibits the m6A demethylase activity of ALKBH5, and the SUMOylation of ALKBH5 can be dynamically and reversibly regulated by the SUMO E3 ligase PIAS4 and the deSUMOylase SENP1.^54^ Additionally, a concurrent study revealed that the acetylation modification on lysine 235 (K235) of ALKBH5, along with its regulatory subunit PSPC1, jointly determines the m6A demethylase activity and oncogenic function of ALKBH5. The K235 acetylation of ALKBH5 enhances its binding recognition to substrate RNA m6A, thus augmenting ALKBH5 removal of m6A modifications on RNA.^55^

In summary, the dynamic regulation of m6A modifications by m6A demethylases plays a balancing role in RNA metabolism. Abnormal expression or dysfunction of m6A demethylases has been shown to be associated with various diseases, including cancer, neurological disorders, and metabolic diseases, highlighting their potential as therapeutic targets in pathological processes.

#### 2.1.3 m6A reader proteins

The dynamic and reversible regulation of m6A modifications is crucially influenced by the interplay between m6A writers and erasers. Various types of m6A methyl readers enhance the diversity of m6A's biological functions. m6A readers include the YTH domain family proteins and the insulin‐like growth factor 2 mRNA‐binding protein (IGF2BP) family.^56^, ^57^ The YTH domain contains approximately 160 amino acids and can bind to single‐stranded RNA. It is highly conserved across yeast, plants, and animals. In human cells, the YTH domain family includes five members: YTHDC1‐2 and YTHDF1‐3. YTHDC1, a nuclear m6A reader, has distinct biological functions compared with cytoplasmic readers. Studies have shown that YTHDC1 can interact with splicing factors SRSF3 and SRSF10,^58^ and then bind to the m6A sites on mRNA, regulating selective splicing of nuclear mRNA. Additionally, the mRNA export factor NXF1 closely associates with SRSF3, promoting the export of m6A‐modified mRNA from the nucleus.^59^ Furthermore, research indicates that YTHDC2 is present in the cytoplasm and not only enhances the translation of m6A‐modified mRNA but also promotes its degradation.^60^ Another YTHDF family members, such as YTHDF1, YTHDF2, and YTHDF3, are located in the cytoplasm and exhibit distinct functions. YTHDF1 promotes mRNA translation, YTHDF2 facilitates mRNA degradation, and YTHDF3 assists in both translation and degradation, though the mechanisms underlying these different functions remain unclear.^61^ Studies have shown that YTHDF1 and YTHDF3 exhibit oncogenic roles in cancers.^61^ Analysis of The Cancer Genome Atlas database revealed that YTHDF1 expression is significantly upregulated in hepatocellular carcinoma (HCC) and positively correlates with pathological stage. Kaplan–Meier analysis indicated that lower levels of YTHDF1 are associated with better survival rates in HCC patients. GO and KEGG pathway analyses of YTHDF1 coexpressed genes suggest that YTHDF1 plays a crucial role in regulating the cell cycle and metabolism of HCC cells.^62^ In lung cancer, YTHDF2 is upregulated and promotes the growth of cancer cells by binding to the m6A‐modified sites in the 3′‐UTR of G6PD, enhancing its translation, and subsequently increasing the pentose phosphate pathway flux, thus driving tumor growth.^61^, ^63^ In CRC, long ncRNA (lncRNA)–GAS5 interacts directly with the WW domain of Yes‐associated protein (YAP), promoting its phosphorylation and ubiquitin‐mediated degradation, thereby weakening YAP‐mediated transcription of YTHDF3. Research shows that YTHDF3 selectively and reversibly binds to m6A‐methylated GAS5, triggering its degradation and forming a GAS5–YAP–YTHDF3 negative feedback loop, thereby promoting CRC progression.^64^, ^65^ Unlike the YTH domain family proteins, IGF2BP family proteins specifically recognize m6A‐modified RNA via their KH domains, stabilizing mRNA rather than promoting its degradation.^56^ The IGF2BP family includes IGF2BP1, IGF2BP2, and IGF2BP3, which are highly expressed in various cancer types and involved in numerous molecular mechanisms. IGF2BP1 and IGF2BP3 are oncofetal proteins produced by tumors and fetal tissues but downregulated in adult tissues.^56^ Recent studies have found that IGF2BP1 binds to the m6A site in the 3′‐UTR of SOX2 mRNA, inhibiting its degradation and thereby promoting the proliferation and metastasis of endometrial cancer cells.^66^ In addition to the aforementioned factors, there is the HNRNPs family, including HNRNPC/G and HNRNPA2/B1. HNRNPA2/B1, as a nuclear m6A reader protein, directly recognizes the RGm6AC motif and regulates RNA selective splicing in a METTL3‐mediated manner.^67^ Furthermore, HNRNPA2/B1 plays a crucial role in promoting the processing of precursor miRNAs. Knocking down HNRNPA2/B1 leads to abnormal alternative splicing in cells, ultimately reducing the production of mature miRNAs.^68^ In summary, m6A reader proteins participate in various aspects of RNA metabolism by recognizing and binding to m6A modifications, including posttranscriptional regulation and cellular responses. Dysregulation of m6A reader proteins is closely associated with the onset and progression of various diseases, indicating their significant role in disease mechanisms and potential as therapeutic targets.

### 2.2 RNA m5C modification

The dynamic modification and demethylation of m5C are primarily mediated by three types of proteins: methyltransferases (writers), demethylases (erasers), and m5C‐recognizing proteins (readers). The term m5C is derived from its presence at the 5th carbon atom of cytosine in RNA.^69^ This modification predominantly occurs in transfer RNA (tRNA) and ribosomal RNA (rRNA), with minor occurrences in mRNA and ncRNA. m5C methylation in mRNA enhances its stability, thereby promoting its expression abundance. Proteins responsible for m5C modification include the NSUN family proteins, which possess the NOL1/NOP2/SUN domain, and tRNA aspartic acid methyltransferase 1 (RDMT1).^70^, ^71^ Specifically, NSUN2 catalyzes m5C modifications in mRNA, while NSUN3 and NSUN4 are localized in mitochondria and catalyze m5C modifications in mitochondrial tRNA and rRNA, respectively. The “erasers” of m5C include ALKBH1 and the TET family proteins.^72^ ALKBH1 removes methyl groups from RNA directly,^73^ while the TET family proteins oxidize m5C to hm5C to facilitate demethylation.^74^ Proteins mediating m5C recognition include Aly/REF export factor (ALYREF) and Y‐box binding protein 1 (YBX1). m5C‐modified mRNA in the nucleus is exported to the cytoplasm under the mediation of ALYREF and NSUN2, recruiting YBX1 to enhance mRNA stability.^30^ Overall, m5C modifications play a crucial role in various aspects of RNA metabolism, including stability, splicing, transport, and translation. These modifications are involved not only in normal cellular functions and developmental processes but also in responses to environmental stress and disease states. Dysregulation of m5C has been closely associated with multiple diseases, including cancer and neurological disorders, highlighting its significant biological importance and potential as a therapeutic target in disease mechanisms.

### 2.3 RNA m7G modification

The m7G modification is one of the most common posttranscriptional modifications, affecting almost all aspects of mRNA, including splicing, polyadenylation, nuclear export, translation, and degradation.^75^ m7G methylation involves the addition of a methyl group to the N7 position of guanine, catalyzed by methyltransferases, typically occurring at the 5′ cap of nascent transcripts.^75^ This essential cap modification plays a pivotal role in regulating RNA lifecycle, transcript stability, and translation.^76^ m7G methylation is present in various molecules, including mRNA, miRNA, tRNA, and rRNA,^76^ and regulates processes such as mRNA transcription, miRNA biogenesis, tRNA stability, and 18S rRNA processing and maturation.^77^ The primary methyltransferase responsible for m7G modification is METTL1 (methyltransferase‐like 1), which targets mRNAs, while WDR4 (WD repeat domain 4) facilitates the binding of the heterodimeric complex to target mRNAs, influencing cancer development by regulating m7G methylation.^78^ The absence of METTL1 reduces m7G‐modified tRNA levels, alters the cell cycle, and suppresses tumorigenicity, while its overexpression promotes oncogenic transformation.^79^ For example, m7G tRNA methylation mediated by the METTL1/WDR4 complex is critical for embryonic stem cell self‐renewal and differentiation,^80^ as well as oncogenic transformation.^80^ Overall, the m7G modification plays a crucial role in RNA stability, nuclear export, translation initiation, and protection against RNA degradation. Dysregulation of m7G modification is closely associated with the development of various diseases, including cancer and viral infections. Understanding the mechanisms of m7G modification is essential for elucidating its role in disease and for developing targeted therapeutic strategies.

### 2.4 RNA N4‐acetylcytidine modification

RNA acetylation in eukaryotes mainly refers to N4‐acetylcytidine (ac4C). ac4C is a conserved, chemically modified nucleoside initially identified in tRNA and rRNA, where it plays a precise role in regulating protein synthesis^81^; RNA acetylation in eukaryotes mainly refers to ac4C. ac4C is a conserved, chemically modified nucleoside initially identified in tRNA and rRNA, where it plays a precise role in regulating protein synthesis.^82^ N‐acetyltransferase 10 (NAT10), a member of the GCN5‐related N‐acetyltransferase family, possesses both acetyltransferase and RNA‐binding domains, catalyzing ac4C modifications in rRNA, tRNA, and mRNA.^83^ Overall, ac4C is a significant RNA modification that plays a crucial role in RNA stability, translation regulation, and intracellular localization.

### 2.5 RNAΨ modification

Ψ is widely present in eukaryotic RNA, including rRNA, mRNA, tRNA, and snRNA. Ψ is derived from uridine through base isomerization, a process known as pseudouridylation.^84^ mRNA pseudouridylation is catalyzed by pseudouridine synthases (PUSs), which alter the chemical structure of uridine nucleotides to form Ψ.^85^PUSs are divided into two categories: independent PUSs that do not require cofactors, and small nucleolar RNA‐dependent PUSs. Research has shown that in Xenopus oocytes, the splicing factor U2AF65 cannot recognize Ψ‐modified polyuridine sequences, leading to defects in precursor mRNA splicing.^86^ Additionally, the absence of H/ACA ribonucleoprotein complex subunit 2 (NHP2), which catalyzes rRNA pseudouridylation, results in defective mitosis in female Drosophila germline stem cells, causing a block in four‐ or eight‐cell cyst formation without affecting the development of normal 16‐cell cysts. Overall, Ψ is a significant RNA modification that plays a crucial role in RNA stability, translation regulation, and intracellular localization.

### 2.6 Inosine modification

Inosine modification occurs through adenosine deamination and is catalyzed by the adenosine deaminase acting on RNA (ADAR) family.^87^ In vertebrates, three ADAR enzymes have been identified: ADAR1, ADAR2 (both catalytically active), and ADAR3 (whose activity remains unreported).^88^ Inosine‐modified RNA is present in both human and mouse oocytes.^89^Inosine preferentially pairs with cytidine, and inosine base mismatches can affect various cellular functions. Inosine modification can recode RNA, influencing codons, splicing sites, RNA secondary structures, and RNA recognition motifs.^90^ Knockout of Adar1 leads to embryonic lethality and multiple organ defects in mice.^91^ During oocyte development, mature oocytes in the GV and MII stages contain more inosine‐modified RNA than oocytes at postnatal day 12, with a specific enrichment in protein‐coding regions, suggesting that inosine modification may influence maternal mRNA stability by altering codon function.^92^ Furthermore, Cnot6l knockout reduces inosine‐modified RNA levels in mouse oocytes, leading to abnormal degradation of maternal mRNA during the MI stage, affecting the maternal‐to‐zygotic transition and regulating embryonic development.^93^

### 2.7 RNA m1A modification

Similar to m6A, the methylation of RNA at the 1st nitrogen atom of adenine is known as m1A modification. m1A is most abundant in tRNA and rRNA, often occurring at the 58th adenosine of tRNA. Under physiological conditions, m1A carries a positive charge, regulating RNA structure and its interactions with proteins. Additionally, m1A modification can block the normal base pairing of A‐T or A‐U, forming Hoogsteen base pairs that alter RNA secondary structure.^94^ m1A is involved in almost all RNA biological processes, including RNA stability, splicing, folding, export, transport, and translation efficiency, with its activity mediated by “writers,” “erasers,” and “readers.” The “writers” of m1A include tRNA methyltransferases (TRMT), with TRMT6/61A functioning in the methylation of tRNA by recruiting TRMT6 to the tRNA and cooperating with TRMT61A, which moves from the cytoplasm to the nucleus to catalyze the modification.^95^ The “erasers” of m1A include demethylases such as ALKBH1, ALKBH3, ALKBH7, and FTO, each acting through different mechanisms. ALKBH family proteins directly remove methyl groups from RNA, while FTO inhibits methyltransferase activity through oxidation.^96^ m1A shares many similarities with m6A, and in alkaline conditions, m1A can convert to m6A. Both modifications can be recognized by some of the same “reader” proteins, such as YTHDF family proteins and YTHDC1.^97^ In summary, m1A significantly impacts RNA stability, translation efficiency, and intracellular localization.

## 3 HEMATOPOIETIC STEM CELLS

**FIGURE 2:**
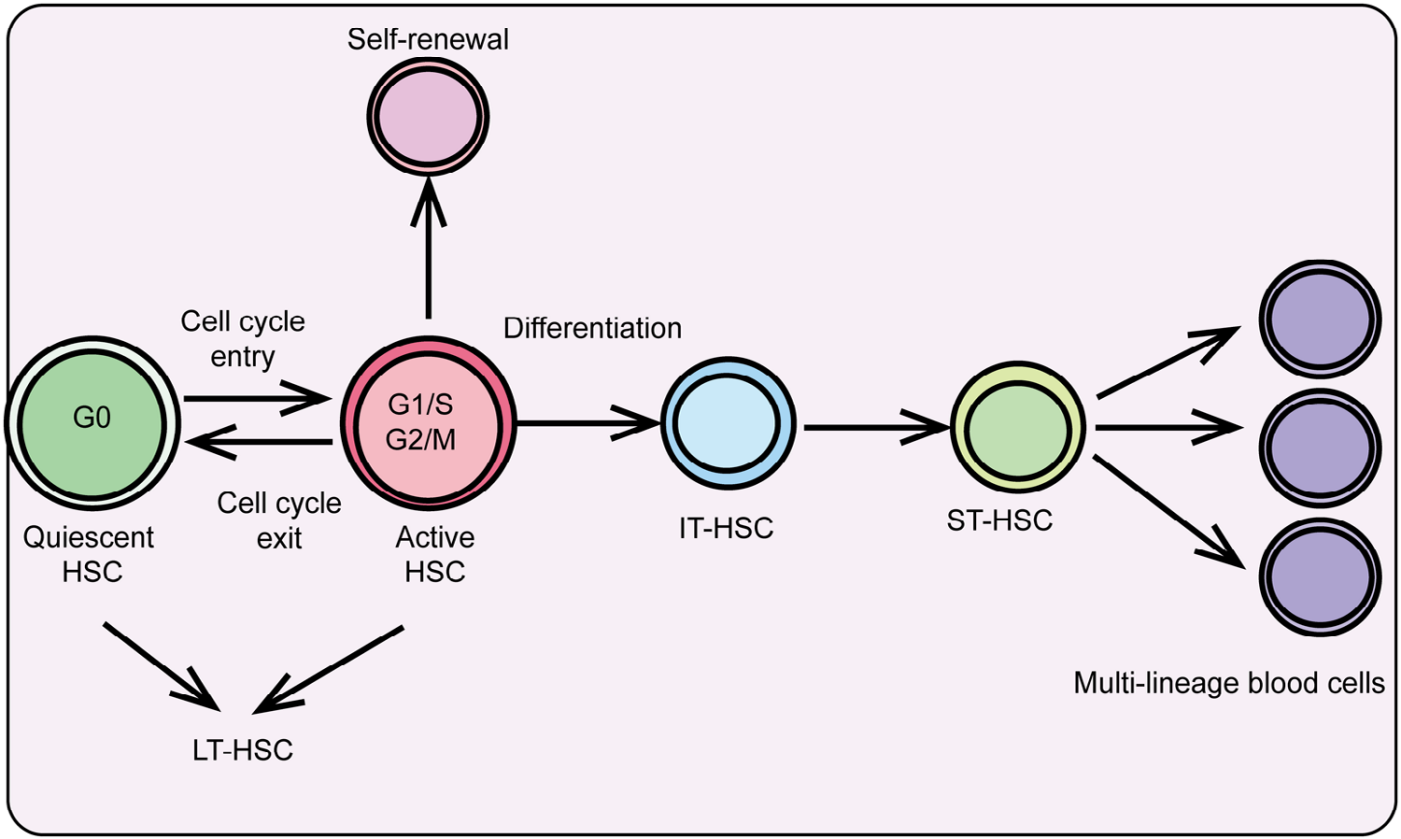
The hierarchical regulation of hematopoietic stem cell differentiation. Under different conditions, hematopoietic stem cells differentiate into various functional cell types. HSC, hematopoietic stem cell; IT‐HSC, intermediate‐term hematopoietic stem cell; ST‐HSC, short‐term hematopoietic stem cell; LT‐HSC, long‐term hematopoietic stem cell.

**FIGURE 3:**
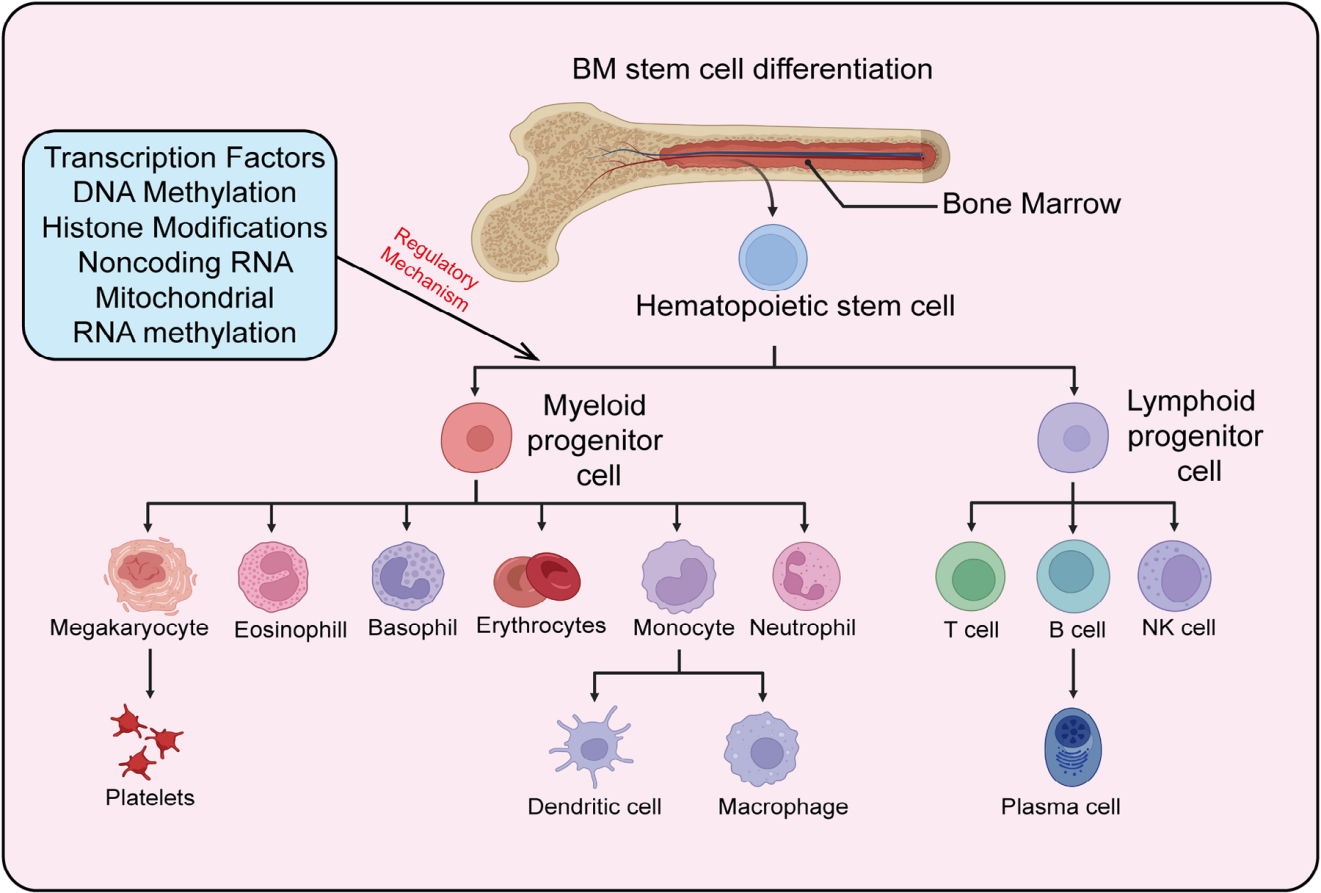
Differentiation of hematopoietic stem cells into diverse functional cell types. The roles of transcription factors, epigenetic modifications (including DNA methylation, histone modifications, RNA modifications, and non‐coding RNAs), as well as mitochondrial functions in regulating hematopoietic stem cell differentiation. T cells, T lymphocytes; B cells, B lymphocytes; NK cells, natural killer cells. The creation of this figure was accomplished with the support of the BioRender platform.

HSCs are a class of cells characterized by their self‐renewal capacity and multipotency, capable of generating various blood cell types in the bone marrow and peripheral blood. They play a crucial role in maintaining hematopoiesis and immune system homeostasis and are extensively used in the clinic to treat a range of hematological disorders, including leukemia, lymphoma, and aplastic anemia. Recent research has also highlighted the significant roles of HSCs in tumor immune evasion, aging, and stem cell therapies, advancing their development in both basic research and clinical applications. Here, we provide a systematic summary of HSCs, including their definition, biological characteristics, differentiation processes, mechanisms regulating differentiation, and particularly the role of epigenetic modifications in controlling HSC differentiation and self‐renewal (Figures 2 and 3).

### 3.1 Definition of HSCs

During hematopoietic development, the formation of HSCs begins in the embryonic yolk sac, then transitions to the aorta–gonad–mesonephros region, exists transiently in the placenta and fetal liver, and finally stabilizes in the bone marrow.^98^ HSCs predominantly reside in the bone marrow cavities of adult mammals, accounting for less than 0.01% of bone marrow cells.^99^ HSCs possess two critical characteristics: high self‐renewal capacity and multipotent differentiation ability. Self‐renewal refers to the ability of stem cells to undergo symmetric division, producing progeny with identical properties to the parent stem cells.^17^ Multipotent differentiation refers to asymmetric division, generating all mature blood and immune cells. Most HSCs remain in a quiescent state (in the G0 phase of the cell cycle) under homeostatic conditions, rarely dividing, with only a small portion (about 5%) actively participating in hematopoiesis at specific stages. This quiescence protects HSCs from external damage, maintaining their genomic integrity and long‐term self‐renewal potential.^100^ However, under acute stress conditions such as bone marrow transplantation, DNA damage, inflammation, acute or chronic infection, and metabolic stress, this protection is rapidly lost. HSCs can be quickly activated, exiting quiescence to enter an active cell cycle for differentiation and division to replenish blood cells in the body. Once the demand is met, homeostasis is restored^101^ (Figure 2).

### 3.2 Differentiation process of HSCs

The continuous production of different lineage mature blood cells by HSCs is termed hematopoiesis, with the human blood system producing approximately 10^11^–10^12^ cells daily. The traditional view posits that HSCs follow a hierarchical differentiation model, progressively differentiating into various blood and immune cells.^102^ During differentiation, HSCs gradually lose their self‐renewal capacity, generating lineage‐restricted progenitor cells, which in turn produce functionally specialized mature blood cells. In the classic hematopoietic hierarchy model, long‐term HSCs (LT‐HSCs) are at the top, giving rise to short‐term HSCs (ST‐HSCs). ST‐HSCs differentiate into multipotent progenitor cells (MPPs), which further differentiate into common myeloid progenitors (CMPs) and lympho‐myeloid progenitors (LMPPs). LMPPs can differentiate into CMPs and common lymphoid progenitors (CLPs); CMPs further differentiate into granulocyte–macrophage progenitors (GMPs) and megakaryocyte–erythroid progenitors (MEPs); CLPs differentiate into mature lymphocytes (T cells, B cells, NK cells); GMPs further differentiate into monocytes, neutrophils, eosinophils, basophils, and macrophages; MEPs ultimately differentiate into erythrocytes and megakaryocytes (producing platelets).^103^ The classical hematopoietic hierarchy is defined based on cell surface markers. With advancing research, perspectives on hematopoietic differentiation are continually updated, with new differentiation models led by lineage‐biased HSCs gaining recognition. According to the classical model, Mkps originate from a cascade differentiation pathway (HSC–MPP–CMP–MEP–Mkp)^104^ (Figure 3). However, with the development of single‐cell techniques (single‐cell transplantation and lineage tracing studies), it has been shown that HSCs can directly differentiate into Mkps, bypassing the intermediate MPP state. The heterogeneity of MPP populations has been redefined based on immune phenotype, cell cycle status, and lineage bias, further categorizing MPPs into MPP1, MPP2, MPP3, and MPP4.^105^ MPP1 is similar to previous ST‐HSCs, capable of producing all mature blood cells; MPP2/3 are myeloid‐biased, producing fewer B and T cells; MPP4 is lymphoid‐biased, producing fewer myeloid cells. Additionally, MPP2 exhibits a platelet bias.^106^ Single‐cell transcriptomics has proposed a new differentiation model where HSC differentiation is a continuous process rather than discrete and intermittent, indicating no clear boundaries between HSCs and hematopoietic progenitors.^107^

### 3.3 Mechanisms regulating HSC differentiation

Adult HSCs maintain blood system homeostasis through the regulation of self‐renewal and multipotent differentiation.^108^ Disruption of this homeostasis can lead to the development and progression of hematological malignancies, such as leukemia, which can arise from excessive self‐renewal of HSCs.^109^ Conversely, insufficient self‐renewal can result in HSC exhaustion and subsequent cell apoptosis, causing conditions such as immunodeficiency or anemia. Currently, HSC transplantation is widely utilized for treating various diseases, including neurological disorders, hematological malignancies, and immune deficiency disorders.^110^ A major clinical challenge is the in vitro induction and expansion of functional hematopoietic stem and progenitor cells (HSPCs) in sufficient numbers, which remains a significant bottleneck and area of focus in basic scientific research. This challenge stems from the incomplete understanding of the mechanisms regulating HSC self‐renewal and multipotent differentiation.^111^ Therefore, in‐depth and systematic studies on the development and regulatory mechanisms of HSCs hold substantial clinical significance. The homeostasis of the blood system is tightly regulated by various lineage‐specific transcription factors, epigenetic modifiers, and cellular metabolic states, which collectively control the activation of lineage‐specific genes and the repression of multipotent genes, thereby maintaining blood system homeostasis^112^ (Figure 3).

#### 3.3.1 Transcription factors

Transcription factors are proteins that bind to specific DNA sequences to regulate gene expression.^113^ Hematopoiesis is an extraordinarily complex process, with many transcription factors playing crucial regulatory roles in normal hematopoiesis. These transcription factors are expressed in a cell‐type and developmental‐stage‐specific manner, controlling the differentiation of HSPCs and the fate determination of hematopoietic cells.^114^ Key transcription factors such as c‐MYC, C/EBPa, and PU.1 are essential for hematopoiesis.^115^ c‐MYC is a critical transcription factor regulating hematopoiesis; its deficiency severely impairs HSC function. C/EBPa is a major regulator of normal hematopoiesis; its knockout in mice leads to ineffective hematopoiesis in progenitor cells, resulting in reduced peripheral blood cells.^116^ PU.1 is involved in hematopoietic regulation, being essential for normal bone marrow and lymphoid development. Variations in PU.1 expression levels are crucial for the differentiation of hematopoietic lineages and the regulation of cell fate.^117^

#### 3.3.2 DNA methylation modifications

Epigenetics refers to stable, heritable changes in gene expression that do not alter the DNA sequence.^118^ Key areas of epigenetics research include DNA methylation, histone modifications, and RNA methylation. DNA methylation and histone modifications primarily regulate gene expression at the transcriptional level, whereas RNA methylation regulates gene expression posttranscriptionally.^119^ Epigenetic modifications dynamically and reversibly regulate HSC self‐renewal, multipotent differentiation, and lineage fate determination.^120^ Comprehensive genome‐wide analyses of epigenetic modifications (such as DNA methylation and histone modifications) during hematopoietic development have been conducted, revealing dynamic changes in epigenetic landscapes that regulate lineage‐specific gene expression and, consequently, cell lineage fate. DNA methylation primarily occurs on cytosine–guanine dinucleotides (CpG sites) and is a reversible epigenetic modification.^121^ Approximately 80% of CpG sites in the human genome are methylated, with most unmethylated CpG sites concentrated in CpG islands located in promoter regions.^121^ DNA methyltransferases (DNMTs) catalyze the conversion of CpG to 5‐methylcytosine (m5C) using S‐adenosylmethionine (SAM) as a methyl donor. The known methyltransferases include DNMT3A, DNMT3B, and DNMT1. DNMT1 mediates maintenance methylation, whereas DNMT3A and DNMT3B are involved in de novo DNA methylation, modifying unmethylated DNA.^122^ The TET protein family (TET1, TET2, and TET3) functions as demethylases, catalyzing the conversion of 5‐mC to hm5C, thus facilitating DNA demethylation. Research has shown that DNA methylation affects genetic stability, gene structure, and gene expression. Promoter CpG island methylation can regulate gene expression, with low promoter methylation being characteristic of gene activation, while high methylation is associated with gene silencing.^123^ Studies have highlighted the significant role of DNA methylation in hematopoietic development. Abnormal activation or inactivation of DNMTs and demethylases can impair HSC development, indicating that DNA methylation levels are crucial in stem cell regulation.^124^ DNMTs play important roles in regulating HSC self‐renewal and differentiation. DNMT1‐mediated methylation is essential for HSC self‐renewal; its knockout leads to hypomethylation in HSCs, resulting in a pronounced bias toward myeloid differentiation and a marked impediment to lymphoid differentiation.^125^ In contrast, DNMT3A knockout leads to increased HSC numbers, impaired hematopoietic reconstitution, and differentiation defects. DNMT3B knockout has minimal impact on HSC function, but combined knockout with DNMT3A causes more severe defects in HSC proliferation and differentiation. Loss of TET family members also promotes HSC self‐renewal and leads to differentiation biases.^126^ TET2, a major TET enzyme affecting hm5C levels in HSCs, is mutated or inactivated in certain cases, leading to increased proliferation of hematopoietic progenitor cells and enhanced self‐renewal.^127^ These findings indicate that HSC homeostasis and self‐renewal require the regulation of DNA methylation. Normally, promoter CpG sites are unmethylated, with only a few dynamically changing. These dynamically changing DNA methylation sites often overlap with binding sites of lineage‐specific transcription factors or enhancers. Methylation of promoters or enhancers can interfere with transcription factor binding and suppress downstream gene expression, thereby regulating HSC differentiation and self‐renewal.

#### 3.3.3 Histone acetylation

The fundamental structural unit of chromatin is the nucleosome, which consists of an octamer of histones wrapped around approximately 147 base pairs of DNA. This histone octamer comprises two copies. The fundamental structural unit of chromatin is the nucleosome, consisting of an octamer of histones wrapped around approximately 147 base pairs of DNA. This histone octamer is composed of two copies each of the core histones H2A, H2B, H3, and H4. The tails of histones extend out from the nucleosome, and posttranslational modifications, such as methylation and acetylation, occur on the tails of histones H3 and H4. The relative activities of histone modification writers and erasers determine the levels of histone modifications, which can be recognized by readers, including chromatin remodelers and transcription factors, to regulate gene transcription and expression.^128^

Histone acetylation promotes chromatin relaxation, allowing transcription factors to bind specifically to DNA binding sites, thereby activating gene transcription and enhancing gene expression.^129^ Conversely, histone deacetylation can lead to chromatin compaction and gene repression. The levels of histone acetylation also impact the biological functions of HSCs.^130^ Abnormalities in histone acetyltransferases can impair the blood system. Knockout of histone deacetylases HDAC1 or HDAC2 affects hematopoiesis, while simultaneous knockout of HDAC1 and HDAC2 results in severe HSC defects, leading to anemia and reduced blood cell counts, indicating functional redundancy between HDAC1 and HDAC2 in hematopoiesis.^131^ SIRT1 (Sirtuin 1), a histone deacetylase, is also involved in the regulation of HSCs. The knockout of SIRT1 impairs the self‐renewal and differentiation capabilities of HSCs.^132^ Similarly, the knockout of SIRT6 (Sirtuin 6) activates the Wnt signaling pathway, promoting HSC proliferation. However, HSCs lacking SIRT6 exhibit impaired self‐renewal capacity in serial competitive transplantation experiments.^133^

#### 3.3.4 Histone methylation

Histone methylation occurs on lysine (K, Lys) and arginine (R, Arg) residues, with histone H3 being the most important, followed by H4. Both lysine and arginine can exist in multiple methylation states. Lysine residues on histones (such as H3K4, H3K9, H3K27, and H3K20) can be monomethylated (me), dimethylated (me2), or trimethylated (me3). Histone methylation influences chromatin conformation and the specific recruitment of downstream effector molecules, thereby regulating the transcription and expression of specific genes.^134^ Histone methyltransferases are a class of enzymes containing the SET domain. The SET1/MLL family complex is the most common H3K4 methyltransferase in mammals. It consists of SET1A/B (KMT2F/G) and MLL1‐4 (KMT2A‐D) as catalytic subunits, along with WRAD (WDR5/RBBP5/ASH2L/DPY30) as the core subunits, which are essential for the methylation activity of the complex. ASH2L and RBBP5 are critical for maintaining the activity of MLL proteins. The SET1/MLL family complex plays a vital role in regulating HSC self‐renewal and progenitor cell expansion. MLL fusion proteins downregulate RUNX1/CBFβ, thereby enhancing the self‐renewal capacity of HSCs. Polycomb repressive complex 2 (PRC2) is a conserved multicomponent transcriptional repressive complex, with core components including EED, SUZ12, RBBP4, EZH1, and EZH2. EZH1 and EZH2 exhibit H3K27 methyltransferase activity, but require EED and SUZ12 for catalytic activity in vitro. While the partial loss of PRC2 caused by heterozygous deletions of EZH1, EZH2, or EED has minimal effects on HSCs, the simultaneous knockout of EZH1 and EZH2 or EED knockout, leading to complete PRC2 depletion, results in HSC exhaustion. This indicates that PRC2 regulates normal HSC function in a dose‐dependent manner. EED knockout leads to HSC exhaustion by disrupting HSC self‐renewal, differentiation, and apoptosis.^135^ There are also reports that PRC2 can regulate NK cell differentiation and function through histone methyltransferase activity.

Histone demethylases, collectively known as the KDM family, all contain the JmjC demethylase domain, except for KDM1. LSD1 (also known as KDM1A) was the first demethylase identified and possesses demethylase activity for both H3K4 and H3K9, thereby repressing or activating its target genes. LSD1 plays a crucial role in hematopoiesis. Complete knockout of LSD1 results in pancytopenia and impairs the self‐renewal and differentiation potential of HSCs. Conditional knockout of LSD1 restricts hematopoietic differentiation and promotes the expansion of HSPCs in the bone marrow by regulating methylation modifications on hematopoietic transcription factors Gfi1 and Gfi1b.^136^ KDM4/JMJD2 is a specific demethylase for H3K9 and H3K36. Conditional knockout of Kdm4a/Kdm4b/Kdm4c in mice demonstrates that KDM4 activity is essential for maintaining HSCs in vivo, as the loss of KDM4 demethylase leads to the accumulation of H3K9me3 at transcription start sites, downregulating genes critical for hematopoiesis.^137^ KDM6A (also known as UTX), a demethylase for H3K27, is part of the compass and SWI/SNF complexes, and plays a key role in regulating aging‐related genes in the hematopoietic system. Histone methylation is dynamically regulated during hematopoietic development. Studies have shown that during HSC differentiation into erythroid progenitors, the levels of H3K4me1, H3K9me1, and H3K27me1 increase in enhancer regions of differentiation‐related genes, suggesting that monomethylation of these histones is involved in the hematopoietic differentiation process.^138^

Bivalent histone methylation refers to the presence of both activating H3K4me3 and repressive H3K27me3 methylation on the same gene, with LT‐HSCs having the largest number of bivalent genes.^138^ As HSCs differentiate, most genes are silenced postdifferentiation and lose H3K4me3, while a few are activated and lose H3K27me3. This suggests that bivalent marks can distinguish specific lineages and remain preserved in parallel differentiation pathways.^139^ A study using iCHIP sequencing to investigate 16 populations during the hematopoietic process focused on four histone modifications: H3K4me1, H3K4me2, H3K4me3, and H3K27ac. Researchers identified 48,415 signal peaks in promoter regions, mapping their dynamic changes throughout differentiation and correlating them with transcriptional changes, illustrating that histone modifications determine the fate of cells.^140^

#### 3.3.5 Noncoding RNA

The Encyclopedia of DNA Elements project has shown that at least 80% of human genomic loci are transcribed into RNA, yet less than 2% of these transcribed regions encode proteins. Among these ncRNAs, those longer than 200 nucleotides are referred to as lncRNAs.^141^ In addition, ncRNAs include miRNAs and circRNAs.^142^, ^143^ miRNAs are small, naturally occurring ncRNAs, approximately 18–25 nucleotides in length, which regulate gene expression by binding to target RNAs, including mRNAs and other ncRNAs, through complementary base pairing.^142^ Hematopoiesis is a complex process regulated by multiple mechanisms, and miRNAs are involved in the directional differentiation of HSCs and the development of hematopoietic cells, including erythropoiesis. They have conserved sequences and a unique circular structure formed through back‐splicing, enabling their stable expression and tissue specificity. With the advancement of transcriptome sequencing technologies, more and more ncRNAs are being discovered and confirmed to play important roles in regulating normal hematopoiesis and HSC self‐renewal.

miRNAs are a class of ncRNAs, only 18–25 nucleotides in length, that regulate gene expression throughout biological evolution. They play a crucial role in the formation of nearly all mammalian tissues. O'Connell and colleagues reported that miR‐125a and miR‐125b are significantly upregulated in LT‐HSCs, and overexpression of miR‐125b significantly enhances bone marrow cell reconstitution capacity, indicating their importance in maintaining HSC self‐renewal.^144^ Additionally, miR‐29a has been reported to regulate the Wnt signaling pathway, thereby maintaining HSC stemness.^145^ Tenedini et al.^146^ reported that miR‐299 is specifically upregulated in megakaryocytes differentiated from human CD34+ hematopoietic progenitor cells in vitro, and overexpression of miR‐299 promotes megakaryocyte and granulocyte differentiation. Ferrari et al.^146^ found that overexpression of miR‐221 and miR‐222 inhibits CD34+ progenitor cell proliferation by downregulating c‐Kit and promotes erythroid differentiation.

Research on the function of lncRNAs in hematopoietic differentiation indicates that they regulate HSC self‐renewal and the differentiation and maturation of progenitor cells at multiple levels, including transcription, posttranscription, and translation. lncRNAs are key components of the gene expression regulatory network and represent important new members of this network. Hematopoiesis is the process by which HSCs gradually differentiate and mature into various functional blood cells. HSCs are a classic type of tissue stem cell with self‐renewal and multipotent differentiation potential. During the classical process of hematopoietic differentiation, HSCs first differentiate into MPPs, which further differentiate into myeloid and lymphoid progenitors, ultimately forming mature cells of various lineages.^147^ As regulatory factors, lncRNAs play essential roles in gene expression and cell fate determination throughout the stages of hematopoietic differentiation. Cabezas‐Wallscheid et al.^148^ constructed a transcriptional profile of HSCs and MPPs in mice and identified 682 lncRNAs specifically expressed in HSCs and MPPs, 79 of which were differentially expressed. However, this study did not specify the functions or mechanisms of these lncRNAs in HSC and MPP differentiation. Subsequently, Luo et al.^149^ used flow sorting technology to isolate HSCs and conducted deep transcriptome sequencing, discovering 323 previously unreported lncRNAs. Comparison of their expression patterns during lineage differentiation revealed that 159 lncRNAs were highly expressed in HSCs, with some exhibiting HSC‐specific expression. These lncRNA genes share epigenetic modification features similar to protein‐coding genes, such as DNA methylation regulating their expression. Knockdown experiments on two lncRNAs specifically expressed in HSCs (lncHSC‐1 and lncHSC‐2) revealed that both play important roles in regulating HSC self‐renewal and lineage differentiation. ChIRP‐seq results of lncHSC‐2 showed that it binds to the bHLH site of the hematopoietic transcription factor E2A and is enriched with other hematopoietic transcription factors (Erg, Fli1, Meis1, Pu.1, etc.) in the surrounding regions. The binding sites of lncHSC‐2 are also enriched with H3K4me3/H3K27ac histone modifications, suggesting that lncHSC‐2 may regulate target gene expression by recruiting key transcription factors through altering their epigenetic states.^149^ These two studies, based on transcriptomics analysis, identified potential lncRNAs that regulate the quiescent and differentiated states of HSCs, providing an important resource for future research.

In addition to using transcriptomic data to screen for lncRNAs that potentially regulate HSC quiescence and differentiation, studies have also explored the roles of lncRNAs involved in other tissues and organs in hematopoietic differentiation. For example, the imprinted lncRNA gene H19, which regulates embryonic development, has also been shown to play a key role in maintaining HSC quiescence in mice. Conditional knockout of the imprinted control region upstream of maternal H19, also known as the H19 differentially methylated region (H19‐DMR), activates the Igf2‐Igf1r pathway, releases FoxO3 (Forkhead box O3)‐mediated cell cycle arrest, and forces quiescent HSCs into the cell division cycle, leading to HSC exhaustion. Under physiological conditions, this pathway is suppressed by miR‐675, which is encoded by an exon of the H19 gene.^150^ The metastasis‐associated lung adenocarcinoma transcription 1 (Malat1) is a highly conserved lncRNA across species. Malat1 is highly expressed in early HSPCs (Lin‐Rhodamine^low^ Hoechst^low^) in the mouse bone marrow and is downregulated in more differentiated progenitor cells (Lin‐Rhodamine^bright^ Hoechst^low^), suggesting that Malat1 may maintain the undifferentiated state of HSPCs. During the differentiation of erythroid myeloid lymphoid (EML) cells induced by all‐trans retinoic acid (ATRA), Malat1 expression is downregulated by P53, which binds to its promoter, thereby inhibiting cell proliferation and indirectly promoting EML cell differentiation.^151^ However, the role of Malat1 in HSPCs and the mechanism by which it regulates their proliferation remains unclear. Overall, ncRNAs play a crucial role in regulating HSCs, contributing to their self‐renewal, differentiation, and functional maintenance. Abnormal expression of ncRNAs has been associated with various hematological disorders, including leukemia and lymphoma, underscoring their importance in the regulation of HSC function and providing new insights for the diagnosis and treatment of related diseases.

#### 3.3.6 Mitochondrial regulation

HSCs maintain blood system homeostasis by regulating cellular metabolism, with mitochondria being a major energy source for cellular metabolism.^152^ This highlights the crucial role of mitochondria in blood system homeostasis. HSCs in a quiescent state maintain a low metabolic rate, relying primarily on glycolysis for energy.^153^ Quiescent HSCs exhibit low mitochondrial metabolic activity, low mitochondrial membrane potential, low oxidative phosphorylation (OXPHOS), high mitochondrial autophagy, and low mitochondrial reactive oxygen species (ROS) levels. Upon differentiation, HSCs exit the quiescent state and enter the cell cycle, shifting their energy source from glycolysis to mitochondrial OXPHOS, accompanied by mitochondrial activation.^154^ This results in increased mitochondrial quality, membrane potential, and ROS levels, with reduced mitochondrial autophagy. There is a significant metabolic difference between quiescent and active HSCs.^155^ Selective degradation of mitochondria through mitochondrial autophagy is critical for maintaining blood system homeostasis; under normal conditions, mitochondrial autophagy removes damaged mitochondria and reduces ROS production. HSCs that cannot undergo autophagy accumulate damaged mitochondria and increased ROS levels, leading to continuous activation of HSCs. When ROS levels exceed cellular antioxidant defenses, HSCs undergo aging or cell death. The deficiency of Atg7 (autophagy‐related gene 7) leads to mitochondrial metabolic dysregulation, impairing HSC quiescence and resulting in HSC exhaustion.^156^ Cellular autophagy maintains normal mitochondrial status to regulate the metabolic rate of HSCs and sustain their quiescence.^156^ The interaction between mitochondria and lysosomes is also crucial for maintaining HSC homeostasis. Quiescent HSCs contain numerous active lysosomes, and lysosomal activity is essential for regulating the quiescent state of HSCs.^157^

## 4 LEUKEMIA STEM CELLS

**FIGURE 4:**
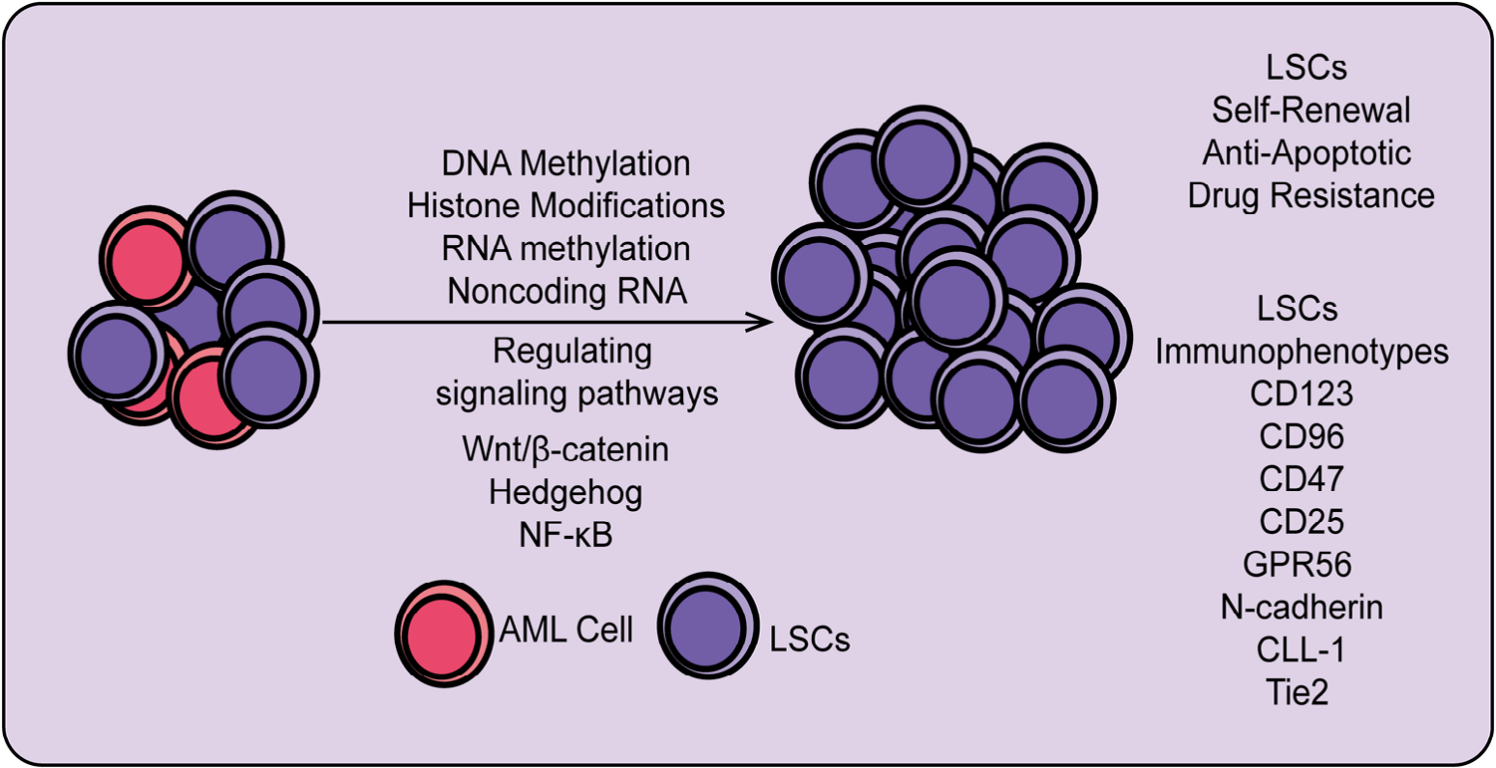
Mechanisms regulating the self‐renewal and differentiation of leukemia stem cells. This includes the characteristics of leukemia stem cells and various regulatory mechanisms, such as transcription factors, epigenetic modifications (including DNA methylation, histone modifications, RNA modifications, and noncoding RNAs). LSC, leukemia stem cell; AML, acute myeloid leukemia; CD123, interleukin‐3 receptor subunit alpha (IL‐3Rα); CD47, cluster of differentiation 47; CD25, interleukin‐2 receptor alpha chain (IL‐2Rα); GPR56, G protein‐coupled receptor 56; CCL‐1, chemokine (C‐C motif) ligand 1; Tie2, tyrosine kinase with immunoglobulin‐like and EGF‐Like domains 2.

Overall, LSCs represent a specific type of stem cell in leukemia, characterized by their self‐renewal capacity and multipotent differentiation potential. They play a central role in the initiation, progression, and drug resistance of leukemia. LSCs contribute to the persistence and relapse of leukemia due to their ability to evade chemotherapy and targeted therapies. While similar to normal HSCs, LSCs exhibit significant alterations in intracellular signaling pathways and gene expression regulation, leading to aberrant hematopoiesis and functional dysregulation. Research indicates that LSCs are distinguished by specific surface markers, transcription factors, and aberrant activation of signaling pathways. Understanding the biological characteristics and molecular mechanisms of LSCs is crucial for developing effective therapeutic strategies and overcoming leukemia relapse. Recently, novel therapies targeting LSCs, including targeted drugs, immunotherapy, and gene editing technologies, are becoming important research directions in leukemia treatment. Here, we systematically summarize the origin, biological characteristics, immune markers, signaling pathways regulating LSC self‐renewal, and particularly the roles and mechanisms of epigenetic modifications (including RNA modifications and ncRNAs) in LSC self‐renewal (Figure 4).

### 4.1 Origins of LSCs

Understanding the origins of LSCs is critical for developing targeted therapies. However, numerous studies suggest that the origins of LSCs are not definitively established. Some research indicates that LSCs may originate from normal HSCs, while the clonal evolution of abnormal hematopoietic stem/progenitor cells is a key mechanism in leukemia development and progression.^158^ HSCs, as adult stem cells in the blood system, have the capacity for long‐term self‐renewal and differentiation into various mature blood cells, capable of asymmetric division into preleukemic HSCs and progenitor cells.^27^ Preleukemic HSCs undergo secondary mutations to become LSCs, which accumulate and undergo malignant transformation, leading to the accumulation of functionally impaired leukemia progenitor cells in the bone marrow or other hematopoietic organs.^159^ Such transformation events confer survival and proliferation advantages to leukemia cells relative to normal HSCs, resulting in the suppression and failure of normal hematopoiesis.^160^ Additionally, the similarity in immune phenotypes (e.g., CD34+CD38‐) between LSCs and HSCs provides evidence that LSCs may originate from HSCs. LSCs may also arise from relatively early myeloid progenitor cells.^161^ CD90 is a cell surface glycoprotein initially identified as a differentiation marker in the mouse brain and thymus.^162^ Research by Han et al.^163^ found that a subset of human umbilical cord blood (hUCB) LIN−CD34+CD38−CD90− contains non‐HSC MPPs, which were inferred to be the origin of AML. Consistent with this hypothesis, Geoffrey Brown et al.^164^ demonstrated that preleukemic mutations occur within clonal HSC populations, ultimately leading to the development of AML MPP LSCs. Another hypothesis posits that LSCs may originate from mature leukemia cells, suggesting that these cells acquire stem cell characteristics due to certain influences, activating stem cell‐related pathways.^165^ Overall, the origins of LSCs remain unclear, but future research may use these potential sources as entry points to identify strategies for targeting LSCs (Figure 4).

### 4.2 Biological characteristics of LSCs

LSCs represent a critical cell population in the initiation and progression of leukemia, exhibiting distinct biological characteristics. First, LSCs possess self‐renewal capabilities, enabling them to maintain their population and drive leukemia's persistence through asymmetric division. Second, LSCs exhibit pluripotency, allowing differentiation into various leukemia cell types, contributing to the heterogeneity and complexity of tumors. Drug resistance is a key feature of LSCs; compared with regular leukemia cells, LSCs demonstrate greater resistance to chemotherapy and radiotherapy.^166^ This resistance is primarily due to their relative quiescence or low proliferation state and the expression of high levels of drug efflux pumps and antiapoptotic proteins. Consequently, traditional treatment methods struggle to completely eradicate LSCs, leading to disease relapse.^159^ Additionally, LSCs show significant environmental adaptability. They interact with stromal cells and other supportive cells in the bone marrow microenvironment to obtain essential survival and proliferation signals. For instance, LSCs can closely adhere to bone marrow stromal cells via adhesion molecules, thus evading immune surveillance and drug effects. At the molecular level, the biological properties of LSCs are closely linked to specific genes and signaling pathways.^167^ Research has identified that genes such as HOXA9, MEIS1, and BCL‐2 are highly expressed in LSCs, promoting their self‐renewal and survival.^168^ Moreover, signaling pathways such as Wnt, Notch, and PI3K/AKT are abnormally active in LSCs, supporting their proliferation and antiapoptotic capabilities.^169^ Overall, LSCs possess unique biological characteristics, including self‐renewal, pluripotency, drug resistance, and environmental adaptability, which play a central role in the occurrence and recurrence of leukemia. Understanding these features is crucial for developing targeted therapeutic strategies and ultimately achieving a cure for leukemia.

#### 4.2.1 Self‐renewal

Self‐renewal is a fundamental characteristic of all stem cells and is one of the most notable features of LSCs.^170^ The biological properties of LSCs share many similarities with HSCs. HSCs are renowned for their proliferation and self‐renewal capabilities, responsible for maintaining the hematopoietic system and residing in the bone marrow.^171^ LSCs have a stronger self‐renewal capacity compared with HSCs and exhibit enhanced cell expansion ability, contributing to the high relapse rate observed after acute AML chemotherapy.^172^ Various signaling pathways regulate the self‐renewal of LSCs, including the Wnt/β‐catenin and Notch signaling pathways.^173^

#### 4.2.2 Antiapoptotic properties

In normal cells, the complex interactions between proapoptotic and antiapoptotic proteins are tightly regulated, with their relative balance determining cellular responses to stress.^174^ An important reason for the high relapse rate in leukemia is the upregulation of antiapoptotic proteins (such as BCL‐2 and BCL‐xL), which prevents apoptosis and thereby promotes the survival of LSCs during treatment.^175^ NF‐κB is a nuclear transcription factor that regulates tumor growth by inducing the expression of its target genes. The BCR–ABL fusion gene in chronic myelogenous leukemia (CML) acts as an antiapoptotic gene, activating various signaling pathways such as PI3K/AKT, JAK/STAT, RAS, and NF‐κB.^176^ The expression of the antiapoptotic protein myeloid cell leukemia 1 has also been linked to NF‐κB activation.^177^ These findings indicate that the expression of antiapoptotic genes in leukemia is related to NF‐κB. Thus, theoretically, targeting NF‐κB activity to reduce antiapoptotic gene expression and induce apoptosis in LSCs could achieve targeted eradication of LSCs.^178^

#### 4.2.3 Drug resistance

LSCs with the CD34+CD38− phenotype exhibit resistance to chemotherapeutic agents, which may contribute to minimal residual disease.^179^ Although the rapid proliferation of differentiated leukemia cells results in adverse clinical outcomes, the LSCs themselves are largely quiescent.^180^ Quiescent LSCs remain in the G0 phase of the cell cycle, making it challenging for conventional chemotherapy, which targets rapidly dividing cancer cells, to eradicate them. The drug resistance of LSCs results in the inability of current treatments to completely eliminate them, leading to a high relapse rate after AML therapy.^181^ Inducing LSCs to exit the quiescent state and enter the cell growth cycle could enhance their sensitivity to chemotherapy, representing an effective therapeutic strategy for eradicating LSCs.

#### 4.2.4 Microenvironment of LSCs

The bone marrow microenvironment supports normal hematopoiesis through the secretion of various growth factors and physical interactions with HSCs and progenitor cells.^182^ Given that LSCs share stem cell‐like characteristics with normal HSCs, they are also likely regulated within the bone marrow microenvironment.^183^ Notably, during the formation of AML, the bone marrow microenvironment contributes to leukemia development through interactions with LSCs. LSCs, while residing in the bone marrow microenvironment, can reciprocally alter this environment, making it more conducive to their survival and less favorable for HSCs.^184^ The bone marrow microenvironment thus acts as a sanctuary for LSCs, aiding their evasion of chemotherapy and immune escape. If drugs can be designed to accurately identify and eliminate LSCs within the bone marrow microenvironment or modify the environment to be unfavorable for LSC survival without affecting normal hematopoiesis, the treatment of AML and prevention of disease relapse could become more manageable.^185^

#### 4.2.5 Immunophenotypes associated with LSCs

The gene expression profile of LSCs, which includes genes specifically expressed by LSCs, is frequently used for risk stratification and prognostic assessment in AML.^186^ During various stages of hematopoietic differentiation in AML, mutations in relevant genes can result in different immunophenotypes for LSCs, thus allowing for the diagnosis and differentiation of leukemia with unclear lineage by identifying these immunophenotypes.^187^ LSCs exhibit stem cell‐like properties and have similar immunophenotypes to normal HSCs, such as CD34+CD38−. However, LSCs also show higher expression of additional markers, such as CD123, CD96, CD47, CD25, G‐protein coupled receptor 56 (GPR56), C‐type lectin‐like receptor‐1 (CLL‐1), interleukin 1 receptor accessory protein (IL1RAP), N‐cadherin, and recombinant TEK tyrosine kinase (Tie2).^188^ CD123, the alpha subunit of the interleukin‐3 (IL‐3) receptor, is highly expressed on LSCs but is not found on normal HSCs, making it a unique marker for leukemia. CD123 can distinguish between normal HSCs, which show almost no CD123 expression, and CD123‐positive LSCs within the CD34+CD38− population. High levels of CD123+ LSCs are negatively correlated with chemotherapy outcomes and prognosis in AML patients, with high CD123 expression at diagnosis serving as an independent prognostic indicator.^189^ Therefore, CD123 may be an important marker for assessing treatment response and prognosis in clinical leukemia management.

CD96, a member of the immunoglobulin superfamily and a novel immune checkpoint receptor, plays a crucial role in antitumor immune responses. CD96 is expressed on LSCs but at lower levels on normal CD34+CD38− cells. In some patients with poor prognosis, CD96 expression can be as high as 90%. Studies on surface molecules of LSCs in children with acute leukemia have shown that CD96 is predominantly expressed in AML, with lower expression in acute lymphoblastic leukemia (ALL).^190^ CD96+ patients tend to have a lower complete remission rate after induction chemotherapy and higher infection and relapse rates compared with CD96− patients. This suggests that CD96+ leukemia patients are more prone to relapse postchemotherapy, indicating CD96 as a potential new prognostic marker.^191^ CD47, also known as integrin‐associated protein, is a transmembrane protein widely expressed on various cell surfaces and is highly expressed on LSCs. Its primary function is to provide an antiphagocytic signal.^192^ High CD47 expression on LSCs enables them to evade phagocytosis and elimination.^193^ Research by Irving L Weissman et al. in 2009 found that CD47 is overexpressed on LSCs derived from AML patients, and CD47 inhibits phagocytosis by interacting with inhibitory receptors on phagocytes, facilitating AML progression. Increased CD47 expression correlates with reduced overall survival in adult AML patients and is an independent factor for poor prognosis.^194^ Additionally, studies using lentiviral vectors to deliver CD47‐siRNA into AML‐derived LSCs demonstrated effective inhibition of LSC proliferation and promotion of apoptosis, highlighting CD47 as a valuable target for leukemia treatment and prognostic prediction.^195^ GPR56 is an adhesion molecule that interacts with the bone marrow niche through binding to type III collagen.^196^ This suggests that GPR56 may play a role in the interaction between LSCs and the bone marrow microenvironment. A multicenter Phase III trial found that GPR56 is highly expressed in AML patients with NPM1 and FLT3 mutations, and its high expression is associated with poor prognosis in AML patients.^197^

N‐cadherin, a classic cadherin in the cadherin superfamily, and Tie2, a receptor tyrosine kinase primarily expressed on endothelial cells and significantly upregulated in tumor neovascularization, are also relevant.^198^ A study by Zhi et al.^199^ analyzed the proportion of CD34+CD38−CD123+ LSCs coexpressing N‐cadherin or Tie2 before and after chemotherapy, finding that chemotherapy enriches the population of N‐cadherin and Tie2 positive CD34+CD38−CD123+ LSCs. Therefore, N‐cadherin and Tie2 may also serve as potential markers for identifying LSCs. CD25, the alpha chain of the IL‐2 receptor, is primarily expressed on normal T cells and induces their proliferation and differentiation. It is also highly expressed on LSCs.^200^ CLL‐1, a type II transmembrane glycoprotein, plays a key role in immune regulation as an inhibitory receptor and is expressed in over 90% of AML patients’ malignant cells but almost not in HSCs.^201^ Similarly, IL1RAP, which shares similarities with CLL‐1, is upregulated in the LSCs of most AML patients and is not expressed in normal HSCs.^202^ Overall, the diverse immunophenotypes highly expressed on LSCs provide more avenues for basic research on LSCs and offer additional options for clinical targeted therapy in AML.

### 4.3 Signaling pathways regulating self‐renewal of LSCs

Several signaling pathways regulate LSCs in the body, each with distinct regulatory roles. Current research has identified several key pathways associated with LSCs, including JAK/STAT, Wnt/β‐catenin, PI3K/AKT, NF‐κB, Hedgehog, and RAS pathways. Among these, the Wnt, Hedgehog, and NF‐κB pathways have been particularly implicated in the self‐renewal and differentiation of LSCs.^27^, ^203^, ^204^ Here, we focus on summarizing the roles of the Wnt, Hedgehog, and NF‐κB pathways in the self‐renewal of LSCs (Figure 4).

#### 4.3.1 Wnt signaling pathway

The Wnt/β‐catenin pathway is a highly conserved signal transduction cascade that plays crucial roles in both normal development and disease states in humans.^205^ Among the four types of Wnt pathways, the Wnt/β‐catenin pathway is classical and primarily involved in gene transcription regulation and activation of nuclear target gene expression. This pathway is often found to be hyperactivated in various cancers and is particularly involved in the self‐renewal and proliferation of LSCs.^206^ β‐Catenin, a central effector and key molecule in Wnt signaling, is critical for LSC self‐renewal, tumorigenesis, progression, relapse, and drug resistance. Theoretically, inhibiting this pathway to suppress β‐catenin expression could potentially reduce or even eliminate the self‐renewal capability of LSCs.^207^ Thus, β‐catenin is considered a potential target for reducing LSC self‐renewal and thereby eliminating LSCs.

#### 4.3.2 Hedgehog signaling pathway

The Hedgehog signaling pathway plays a significant role in embryonic development, maintaining adult stem cells, and regulating cell proliferation and differentiation.^208^ In addition to its roles in normal embryogenesis and tissue homeostasis, abnormalities in the Hedgehog pathway also affect the survival of leukemia‐associated cells. The Hedgehog pathway can be activated through two main routes, with a focus on the canonical pathway here.^209^ The canonical pathway involves three ligands—Indian Hedgehog, Desert Hedgehog, and Sonic Hedgehog—and two receptors—Smoothened (SMO) and Patched (PTCH). In the absence of ligands, PTCH inhibits SMO, whereas in the presence of ligands, ligand binding to PTCH relieves this inhibition, allowing SMO to activate glioma‐associated oncogene homolog (GLI) through phosphorylation.^210^ Activated GLI binds to target DNA in the nucleus, leading to the expression of specific genes involved in cell cycle induction, antiapoptosis, and cell differentiation. As a crucial node in the Hedgehog pathway, inhibition or reduction of SMO can decrease the number of LSCs and impair their self‐renewal capability.^211^ Therefore, targeting SMO to reduce its expression could potentially improve the cure rate for leukemia.

#### 4.3.3 NF‐κB signaling pathway

The NF‐κB signaling pathway is persistently activated in various leukemia cells, especially in LSCs.^212^ NF‐κB is a vital transcription factor involved in cell survival, proliferation, and differentiation. It is expressed at lower levels in normal HSCs but is significantly overexpressed in LSCs.^213^ The overactivation of NF‐κB in LSCs induces the expression of antiapoptotic genes, blocking LSC apoptosis and contributing to the persistence of AML, resulting in decreased treatment efficacy and increased relapse rates. Inhibiting the abnormal expression of NF‐κB during AML progression and downregulating antiapoptotic pathways to restore normal apoptosis could potentially impede the proliferation and differentiation of LSCs, leading to a reduction in their number.^213^ In summary, these distinct signaling pathways regulate various downstream target genes and play crucial roles in the self‐renewal of LSCs.

### 4.4 Noncoding RNA regulating self‐renewal of LSCs

ncRNAs, including lncRNAs, microRNAs miRNAs, and circRNAs, have been reported to regulate the self‐renewal, proliferation, and leukemogenesis of LSCs through their abnormal expression. For instance, Gao et al.^214^ identified that lncRNA HOTAIR is significantly upregulated in LSCs compared with HSCs. Silencing HOTAIR markedly inhibits self‐renewal, proliferation, and colony formation in AML blasts, as well as the self‐renewal of LSCs. Mechanistic studies revealed that HOTAIR promotes LSC self‐renewal by recruiting EZH2‐mediated H3K27me3 to suppress p15 expression.^214^Additionally, LAMP5‐AS1 has been found to bind and regulate the methyltransferase activity of DOT1L, thereby promoting self‐renewal in MLL leukemia cells. The lncRNA ANCR is also highly expressed in LSCs, where it enhances LSC self‐renewal by modulating MYC oncogene expression.^215^ Cui et al.^216^ discovered that LINC00152 is crucial for LSC self‐renewal; knockdown of LINC00152 inhibits colony formation of CD34+ AML cells and increases their chemotherapeutic sensitivity, although the molecular mechanisms regulating LSC self‐renewal remain unclear. Studies have shown that miR‐31‐5p is downregulated in LSCs and significantly upregulated in HSCs. Knockdown of miR‐31‐5p in HSPCs upregulates the downstream target gene FIH, thereby inhibiting the HIF‐1α signaling pathway. Furthermore, overexpression of miR‐31‐5p significantly suppresses the self‐renewal capacity of LSCs, although the precise molecular inhibition remains unclear.^217^ Another study confirmed a significant downregulation of microRNA miR‐30e‐5p in LSCs. Overexpression of miR‐30e effectively inhibits leukemia formation, impairs LSC self‐renewal, and delays leukemia progression. Further research revealed that miR‐30e‐5p directly targets Cyb561, playing a key role in inhibiting LSC self‐renewal.^218^ In AML, miR‐126, aberrantly upregulated due to the CBFB–MYH11 fusion gene, directly targets and regulates the SPRED1/PLK2–ERK–MYC axis, enhancing MYC activity and improving antileukemia and anti‐LSC effects.^219^ CircFAM193B is significantly upregulated in AML, and its overexpression markedly promotes chemotherapy resistance and AML progression in LSCs. Silencing circFAM193B enhances mitochondrial OXPHOS function and inhibits chemotherapy‐induced ROS accumulation and lipid peroxidation, thus protecting AML cells from oxidative stress‐induced cell death. Mechanistic studies show that circFAM193B interacts with arginine methyltransferase PRMT6, reducing H3R2me2a modification levels, which in turn upregulates the lipid peroxidation factor ALOX15, thereby promoting LSC resistance.^220^ Similarly, circ_0012152 is highly expressed in AML tissues and cells; inhibiting its expression reduces AML cell proliferation and promotes cell death. Further research found that circ_0012152 acts as a molecular sponge for miR‐625‐5p, upregulating the downstream target gene SOX12, which subsequently promotes cell proliferation.^221^ In contrast, hsa‐circ_0003420 is downregulated in LSCs; overexpression of hsa‐circ_0003420 promotes LSC death and inhibits the expression of leukemia tumor stem cell self‐renewal markers. Mechanistic studies found that hsa‐circ_0003420 directly targets and inhibits the mRNA of IGF2BP1, thereby suppressing LSC function.^222^ All the above‐mentioned studies illustrate the diverse functions of ncRNAs in LSC self‐renewal and AML progression.

## 5 THE FUNCTION AND MECHANISM OF M6A MODIFICATION IN NORMAL HEMATOPOIESIS

**FIGURE 5:**
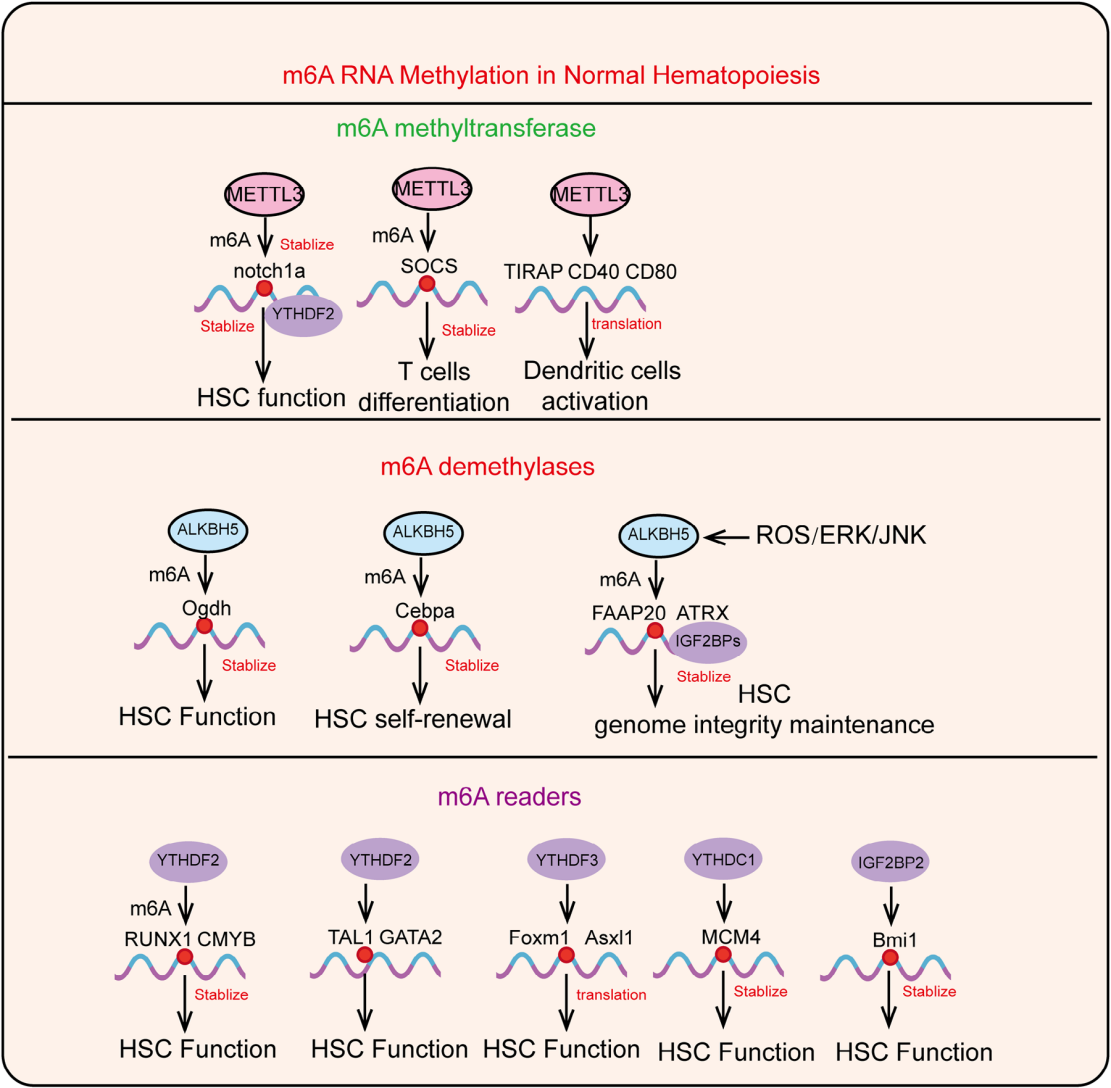
Mechanisms of m6A modification in normal hematopoiesis and the self‐renewal and functional maintenance of hematopoietic stem cells. m6A methyltransferases, demethylases, and reader proteins regulate hematopoiesis by modulating RNA stability, degradation, and translation efficiency. NOTCH1A, neurogenic locus notch homolog protein 1A; SOCS, suppressor of cytokine signaling; TIRA, TIR domain‐containing adapter protein; CD40, cluster of differentiation 40; CD80, cluster of differentiation 80; OGDH, oxoglutarate dehydrogenase; CEBPA, CCAAT/enhancer‐binding protein alpha; FAAP20, Fanconi anemia‐associated protein of 20 kDa; ROS, reactive oxygen species; ERK, extracellular signal‐regulated kinase; JNK, c‐Jun N‐terminal kinase; RUNX1, Runt‐related transcription factor 1; CYMB, cytochrome *b* (possibly referring to a subunit of the cytochrome complex, such as CYB5 or CYB5R3); TLA1, thylakoid lumenal 1 protein; GATA2, GATA‐binding factor 2; FOXM1, Forkhead box protein M1; ASX1, ASXL transcriptional regulator 1; MCM4, minichromosome maintenance complex component 4; BMI1, polycomb complex protein BMI1.

Research into the regulatory functions and mechanisms of m6A modification in HSCs has demonstrated that this epitranscriptomic modification affects HSC biology through multiple pathways. m6A modification primarily influences posttranscriptional processes, including RNA stability, splicing, nuclear export, and translation efficiency, thereby having a profound impact on gene expression.^223^ This modification plays a critical role in the self‐renewal, differentiation, and fate determination of HSCs. During normal hematopoiesis, m6A modification maintains HSC equilibrium by regulating the expression of a range of key genes. For instance, m6A modification affects the expression of genes such as MYC and PTEN, which are crucial for HSC self‐renewal and differentiation.^224^ Additionally, m6A modification regulates signaling pathways such as PI3K/AKT and JAK/STAT to ensure proper HSC function and homeostasis. Furthermore, m6A modification also affects the function of ncRNAs, influencing the fate determination of HSCs. Changes in m6A modification can impact the stability and function of microRNAs and long ncRNAs, thereby indirectly regulating hematopoiesis‐related gene expression.^225^ Overall, m6A modification plays a key role in maintaining HSC self‐renewal, differentiation, and pluripotency through the fine‐tuned regulation of gene expression and signaling pathway activity. Understanding this regulatory mechanism not only enhances our comprehension of hematopoiesis but also provides new potential targets and strategies for treating hematological diseases.^226^ Therefore, here, we systematically summarize the functions and mechanisms of m6A modification regulatory factors in normal hematopoiesis (Figure 5).

### 5.1 Role of m6A MTCs in normal hematopoiesis

To investigate the role of m6A modification in normal hematopoiesis, Zhang et al.^227^ conducted a series of studies using zebrafish. In embryos with METTL3 deletion, normal HSC production was impaired, while endothelial characteristics of blood vessels were significantly enhanced, indicating a blockade of endothelial–hematopoietic transition (EHT). Multiomics analyses revealed that in METTL3‐deficient embryos, the m6A modification levels of a series of arterial endothelial development‐related genes, particularly notch1a, were significantly reduced, while their mRNA levels were markedly increased. These results demonstrate that m6A modification is involved in the balanced regulation of endothelial and hematopoietic gene expression during the EHT.^228^ Additionally, m6A modification maintains the balance of endothelial and hematopoietic gene expression during EHT through YTHDF2‐mediated stabilization of notch1a mRNA, thereby regulating HSC fate.^228^ Lv et al.^229^ confirmed that RNA m6A specific to endothelial cells also plays a significant regulatory role in the development of mouse HSPCs. Besides finding that m6A methylation is crucial for HSPC development during embryogenesis, Yao et al.^230^ explored the roles of METTL3 and METTL14 in the regulation of HSC self‐renewal in adult mouse bone marrow. Their results showed that Mx1–Cre; METTL3^fl/fl conditional knockout mice had an increased number of HSCs in the bone marrow but exhibited significantly impaired reconstitution function.^230^ Furthermore, Cheng et al.^231^ discovered that METTL3 maintains the maturation and terminal differentiation of myeloid and erythroid cells. Conversely, Weng et al.^35^ reported that METTL14 plays a role in suppressing normal myeloid cell production. Thus, while METTL3 and METTL14 are both crucial in normal hematopoiesis, their effects are not identical.^35^ In addition to these animal studies, Vu et al.^231^ knocked down METTL3 in hUCB HSPCs and found that cell proliferation was inhibited, while myeloid differentiation increased. Subsequently, Weng et al. knocked down METTL14 in hUCB HSPCs and observed results consistent with those of Vu et al.^231^ Moreover, m6A modification is also involved in immune regulation. Li et al.^232^ first elucidated the role of m6A modification in maintaining T cell homeostasis in mice. They found that in CD4–Cre; METTL3^fl/fl conditional knockout mice, there was an increase in immature T cells in the spleen and lymph nodes, with METTL3‐deficient naive T cells showing reduced differentiation into Th1 and Th17 cells, but increased differentiation into Th2 cells. Thus, m6A modification is essential for the proper exit of T cells from the immature “progenitor” state and necessary for Th cell differentiation and proliferation in vivo.^232^ Further studies have shown that under normal conditions, METTL3 degrades SOCS mRNA, thereby activating the downstream target gene STAT5, initiating reprogramming of immature T cells, and promoting their differentiation and proliferation. This study focused on m6A‐dependent mRNA decay,^232^ while Furlan et al.^233^ suggested that m6A modification affects multiple stages of RNA life cycle, with reduced m6A levels delaying RNA synthesis, impairing its processing, and upregulating genes and pathways related to RNA decay, thus influencing all RNA dynamics during T cell differentiation. Additionally, Wang et al.^234^ reported the regulatory role of METTL3‐mediated m6A methylation in innate immunity. They found that METTL3 knockout in dendritic cells led to increased translation efficiency of TIRAP, CD40, and CD80 mRNA through m6A methylation. Upregulation of CD40 and CD80 expression aids in antigen presentation and T cell stimulation by dendritic cells, while high expression of TIRAP enhances TLR4/NF‐κB signaling and inflammatory cytokine secretion, ultimately mediating dendritic cell activation and maturation.^234^ Overall, the above studies indicate that m6A methyltransferases play a crucial role in regulating normal hematopoiesis.

### 5.2 The role of m6A demethylases in normal hematopoiesis

As one of the m6A demethylases, ALKBH5 deficiency results in a moderate increase in the number of several progenitor cell populations while impairing the long‐term self‐renewal capacity of HSCs. Mechanistic studies suggest that ALKBH5 may regulate hematopoiesis by reducing the m6A modification of Cebpa and maintaining gene expression levels.^235^ Another study indicates that ALKBH5 regulates mitochondrial ATP production in an m6A‐dependent manner and affects the adaptation of HSPCs. ALKBH5 deficiency upregulates RNA methylation levels and decreases the stability of Ogdh RNA, slowing down the tricarboxylic acid (TCA) cycle, leading to the accumulation of α‐ketoglutarate and its conversion to L‐2‐hydroxyglutarate (L‐2‐HG). L‐2‐HG significantly inhibits in vitro energy production in mouse and human hematopoietic cells, placing HSPCs at a competitive disadvantage.^236^ Additionally, our previous research found that ALKBH5 is critical for genomic integrity maintenance and HSC cell survival. ROS promotes ALKBH5 SUMOylation by activating the ERK/JNK signaling pathway, regulating the demethylase activity of ALKBH5, and thereby modulating the stability of DNA damage repair‐related genes to maintain HSPC genomic stability.^237^ Overall, the above studies indicate that m6A demethylases play a crucial role in regulating normal hematopoiesis.

### 5.3 The role of m6A reader proteins in normal hematopoiesis

In addition to knocking down METTL3 in zebrafish, Zhang et al.^10^, ^238^ independently knocked down YTHDF2 and found that characteristic genes of HSPCs, such as RUNX1 and CMYB, were downregulated, and the number of hematopoietic endothelial cells and HSPCs decreased, indicating that YTHDF2 also plays a crucial role in HSPC development. Moreover, Li et al.^229^ used Mx1–Cre; YTHDF2^fl/fl conditional knockout mice and hUCB HSPCs to investigate the effects of YTHDF2 knockdown. They found that knockout of YTHDF2 in mice and knockdown of YTHDF2 in humans both resulted in an increased number of functional HSCs without interfering with lineage differentiation or promoting abnormal proliferation. Further research revealed that inhibition of YTHDF2 in mouse and hUCB HSCs led to increased expression of several transcription factors critical for self‐renewal, such as TAL1, GATA2, RUNX1, and STAT5, thereby promoting HSC expansion.^229^ Similarly, Wang et al.^239^ obtained comparable results. However, they found that the lack of YTHDF2 enhanced the expression of the Wnt signaling pathway, ultimately increasing HSC regenerative capacity.^239^ In contrast, the impact of YTHDF2 on HSPCs is more limited to the regulation of self‐renewal. In mouse models, Ythdf2 knockout promoted the proliferation of adult HSCs and simultaneously enhanced their self‐renewal ability. Under hematopoietic stress conditions induced by 5‐FU and IR, YTHDF2 deficiency was found to enhance the regeneration and antiapoptotic capacity of adult HSCs. Transcriptomic analyses showed that YTHDF2 regulates HSC function by degrading mRNA of molecules related to the Wnt pathway and antiapoptotic pathways.^239^ A similar study demonstrated that inactivation of YTHDF2 leads to HSC expansion, with HSCs in Ythdf2‐deficient mice undergoing exhaustion after continuous transplantation, accompanied by increased abundance of inflammation‐related transcripts with various m6A modifications and prolonged activation of proinflammatory pathways. Hematopoietic‐specific Ythdf2 deficiency causes progressive myeloid bias, loss of lymphoid potential, HSC expansion, and aging. Ythdf2‐deficient HSCs fail to reconstitute multilineage hematopoiesis.^240^ Our previous research revealed that although YTHDC1 significantly promotes AML progression, its suppression in healthy human‐derived progenitor cells (CD34+) has a minimal impact. In mouse models, Ythdc1 knockout led to complete mortality within 3 weeks, with a significant decrease in various mature cell numbers and nearly undetectable levels of HSCs, presenting a typical acute bone marrow failure phenotype. Bone marrow competitive experiments showed a significant decline in HSC function following Ythdc1 knockout. Interestingly, the phenotype caused by Ythdc1 knockout was more pronounced than that observed in Mettl3 knockout mice, suggesting that Ythdc1 may have non‐m6A‐dependent functions in HSCs.^241^ Our research reveals that YTHDF3 is essential for maintaining HSCs under stress conditions but is dispensable for normal hematopoiesis under homeostatic conditions. Mechanistic studies show that Ythdf3, rather than YTHDF1 and YTHDF2, promotes the translation of Foxm1 and Asxl1, thereby protecting HSC integrity under stress.^242^ Consistent with our findings, another research team discovered that dysfunction of Ythdf3 and Ccnd1 severely impairs HSC reconstitution, resembling the phenotype of Mettl3‐deficient HSCs. Further investigation revealed that Ythdf3 and Mettl3 regulate HSC reconstitution by modulating the m6A RNA methylation of Ccnd1.^243^ IGF2BP2 has recently been reported to be highly expressed in LT‐HSCs, maintaining the stability of m6A‐modified mRNA in LT‐HSCs. Physiological studies using Igf2bp2 knockout mice revealed reduced LT‐HSC numbers, diminished colony‐forming ability, increased apoptosis, loss of quiescence, and severely impaired long‐term hematopoietic reconstitution, confirming the important role of IGF2BP2 in maintaining HSC function. Furthermore, Bmi1 has been identified as a key downstream target of Igf2bp2 in LT‐HSCs; Igf2bp2 deficiency promotes Bmi1 mRNA degradation, thereby relieving the inhibition of Bmi1 on mitochondrial gene expression and ultimately leading to impaired HSC function.^244^ Overall, m6A modification plays a critical role in normal hematopoiesis by regulating RNA stability, splicing, translation, and degradation, which in turn affects the proliferation and differentiation of HSCs and ensures the generation of appropriate blood cell types at various developmental stages. It also contributes to the maintenance and adaptive responses of HSCs and their progenitor cells by modulating the expression of transcription factors and signaling pathways. Additionally, aberrations in m6A modification can lead to disruptions in hematopoiesis and may be associated with various hematological disorders. Understanding the role of m6A modification in normal hematopoiesis is crucial for elucidating its function in pathological conditions and for developing related therapeutic strategies.

## 6 THE FUNCTION AND MECHANISM OF RNA MODIFICATION IN HEMATOLOGIC MALIGNANCIES

RNA chemical modifications play a crucial role in hematological malignancies by regulating RNA stability, transport, splicing, and translation, which in turn affects the biological characteristics of tumor cells. Common RNA modifications include m6A, m5C, ac4C, and m7G. These modifications influence the development and progression of hematological cancers, such as leukemia and lymphoma, by modulating key transcription factors and signaling pathways. Understanding the functions of these modifications in hematological tumors is essential for developing new therapeutic strategies and enhancing existing treatments. Therefore, here we provide a focused summary of the roles and mechanisms of m6A, m5C, ac4C, and m7G in hematological malignancies, particularly in the progression of AML, offering theoretical insights for the treatment of malignant hematological diseases.

### 6.1 The function and mechanism of m6a modification in hematologic malignancies

**FIGURE 6:**
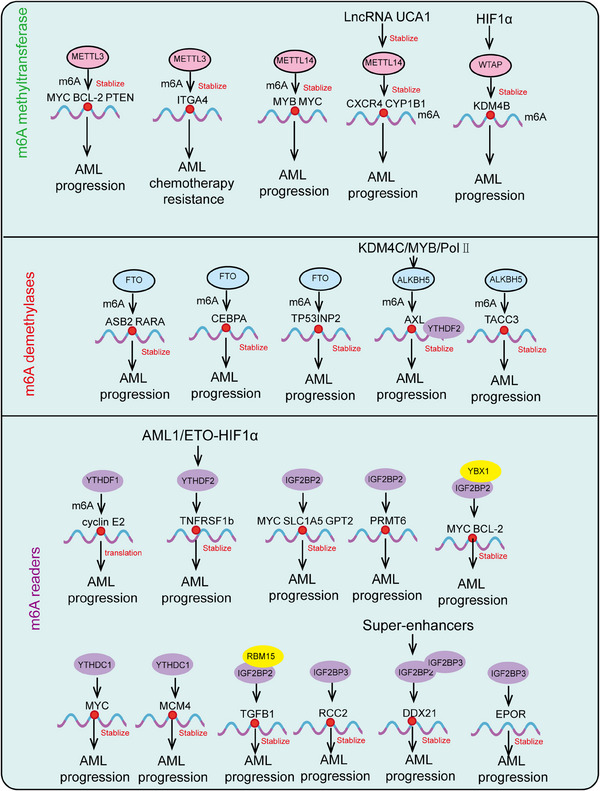
Mechanisms of m6A modification in acute leukemia and the self‐renewal and functional maintenance of leukemia stem cells. m6A methyltransferases, demethylases, and reader proteins participate in the self‐renewal of leukemia stem cells and the progression of AML by regulating RNA stability, degradation, and translation efficiency. MYC, myelocytomatosis oncogene (also known as c‐Myc); TACC3, transforming acidic coiled‐coil‐containing protein 3; CXCR4, C‐X‐C chemokine receptor type 4; KDM4B, lysine demethylase 4B; CEBPA, CCAAT/enhancer‐binding protein alpha; AXL, AXL receptor tyrosine kinase; PRMT6, protein arginine methyltransferase 6; RCC2, regulator of chromosome condensation 2; DDX21, DEAD‐box helicase 21; EPOR, erythropoietin receptor.

Research on the function and mechanisms of m6A modification in hematologic malignancies reveals that this modification exerts significant effects on gene expression, signaling pathways, and cell fate determination by regulating posttranscriptional RNA modifications.^245^, ^246^ Abnormal changes in m6A modification levels are considered key factors in the initiation and progression of hematologic malignancies such as leukemia. Specifically, here we summarize how m6A modification contributes to the progression of malignant hematological diseases, particularly AML, by affecting RNA stability, degradation, and translation efficiency (Figure 6).

#### 6.1.1 The role of m6A MTCs in AML

METTL3 plays a critical role in both normal and malignant hematopoiesis. In normal hematopoiesis, METTL3 maintains the homeostasis of the bone marrow microenvironment for HSCs and suppresses the myeloid differentiation of HSCs.^227^, ^247^ Silencing METTL3 or reducing m6A methylation levels can induce differentiation and apoptosis of bone marrow mesenchymal stem cells, leading to hematopoietic failure.^227^ In AML, METTL3 is overexpressed compared with normal HSCs. Through its m6A methyltransferase function, METTL3 increases the m6A methylation levels of MYC, BCL‐2, and PTEN, thereby upregulating their expression. This action inhibits normal HSC differentiation and induces hematopoietic arrest at an earlier stage.^248^, ^249^ Sang et al.^250^ found that METTL3 knockout induces AML cell differentiation and promotes apoptosis. Furthermore, METTL3 expression is upregulated in AML patients at diagnosis and during relapse, but downregulated upon achieving complete remission, suggesting a potential role of METTL3 in AML drug resistance.^250^ Another study by Li et al.^249^ demonstrated through RNA sequencing of complete remission and relapsed AML patients that METTL3 plays a significant role in AML chemoresistance. Cell experiments confirmed that METTL3 enhances ITGA4 mRNA stability in an m6A‐dependent manner, upregulating ITGA4 protein expression, which increases AML cell adhesion and migration, promoting the homing and engraftment of AML cells in the bone marrow microenvironment, and ultimately leading to chemoresistance.^249^ METTL14 is highly expressed in AML cells and regulates the stability of MYB and MYC mRNA through m6A methylation. This regulation enhances the self‐renewal capacity of LSCs/leukemia‐initiating cells (LSCs/LICs) and contributes to the oncogenic potential of AML.^35^ Lei et al.^251^ discovered that METTL14 oncogenic role in AML is positively regulated by lncRNA. Through in vivo and in vitro experiments, they found that lncRNA UCA1 binds to METTL14, promoting its stability and upregulating CXCR4 and CYP1B1 protein levels, which enhances AML cell proliferation and progression.^251^ This study links METTL14 with lncRNA, suggesting a new molecular mechanism for AML development and providing new insights into AML cell proliferation and progression. Another study connected METTL14 with miRNA and validated that miR‐1306‐5p downregulates METTL14 expression, thereby decreasing MYB and MYC m6A methylation levels, reducing the stability of their mRNA transcripts, and promoting AML cell apoptosis.^252^ This negative regulatory role of miRNA in AML offers new strategies for future AML treatments. These studies indicate that, beyond regulating mRNA, m6A methylation also involves the regulation of lncRNA and miRNA. METTL16 has been identified as essential for the survival of AML cells. It is overexpressed in human AML as well as LSCs and LICs. Knockout of METTL16 significantly inhibits AML initiation/development and maintenance and markedly impairs LSC/LIC self‐renewal, with moderate effects on normal hematopoiesis in mice. Mechanistic studies show that METTL16 exerts its oncogenic effects by promoting the expression of branched‐chain amino acid (BCAA) transaminase 1 (BCAT1) and BCAT2 in an m6A‐dependent manner, thereby reprogramming BCAA metabolism in AML.^226^ In addition to its role in regulating ncRNAs, another study by Shao et al. revealed the role of the m6A methyltransferase WTAP in histone modification regulation.^253^ This study confirmed that the transcriptional activator HIF1α binds to WTAP to positively regulate the m6A methylation level of KDM4B mRNA transcripts, upregulating KDM4B protein expression. This upregulation inhibits H3K9me3 activity, thereby promoting AML cell proliferation.^254^ This finding offers new insights into AML development and progression from the perspective of WTAP interaction with histone methylation modifications (Figure 6). Overall, the above studies indicate that m6A methyltransferase play a crucial role in the progression of AML.

#### 6.1.2 The role of m6A demethylases in AML

Studies have shown that FTO is overexpressed in AML with FLT3–ITD and NPM1 mutations. This overexpression downregulates m6A methylation levels of ASB2 and RARA mRNA, leading to decreased ASB2 and RARA protein expression. Consequently, this diminishes the sensitivity of AML cells to ATRA, thus promoting cell proliferation and AML progression.^255^ Therefore, targeting FTO with inhibitors in combination with ATRA holds substantial potential for AML treatment. Previous research has reported that FTO inhibitors enhance m6A methylation of CEBPA mRNA, reducing CEBPA protein levels and ultimately inhibiting AML cell proliferation.^256^ Thus, targeting the FTO/m6A/CEBPA pathway could also be a viable strategy for AML therapy. In AML subtypes with NPM1 mutations, which exhibit high autophagic activity, the specific mechanisms maintaining this high level of autophagy remain unclear. Huang et al.^257^ confirmed that FTO plays a critical role in maintaining high autophagic activity in NPM1+ AML. FTO lowers the m6A methylation level of TP53INP2 mRNA, thereby upregulating TP53INP2 protein expression. This enhancement promotes interactions with autophagy‐related proteins such as microtubule‐associated protein 1 light chain 3 (LC3) and ATG7, thus increasing autophagic activity in NPM1+ AML cells and inducing cell survival and proliferation.^257^ Hence, targeting and inhibiting FTO and TP53INP2 could improve therapeutic precision and may be particularly effective in NPM1 AML with high FTO expression, potentially translating into clinical applications. These studies suggest that FTO role in AML involves various cellular regulatory pathways, providing a theoretical basis for the development of FTO inhibitors. Wang et al.^258^ found that during the leukemogenesis of AML, ALKBH5 expression is regulated by chromatin state changes and is essential for maintaining LSC function. Mechanistic studies revealed that lysine‐specific histone demethylase 4C (KDM4C) increases ALKBH5 gene chromatin accessibility by downregulating H3K9me3 expression, promoting MYB and Pol II recruitment, thereby sustaining high ALKBH5 expression. AXL is one of the targets of ALKBH5; ALKBH5 regulates AXL mRNA stability in an m6A‐dependent manner through YTHDF2, thus promoting AML cell proliferation and maintaining AML LSC function. This provides a theoretical foundation for targeting ALKBH5 to eliminate LSCs and overcome AML resistance.^258^ In another study, Shen et al.^259^ identified TACC3 as another target of ALKBH5. ALKBH5 downregulates P21 and upregulates MYC protein levels by modulating TACC3 mRNA stability, thereby promoting AML cell proliferation and LSC self‐renewal, which is associated with poor outcomes in AML patients. Therefore, targeting ALKBH5 and TACC3 holds promise as a therapeutic approach for AML (Figure 6). Overall, the above studies indicate that m6A demethylases play a crucial role in the progression of AML.

#### 6.1.3 The role of m6A reader proteins in AML

Recent research has shown that YTHDF1 is overexpressed in human AML samples and significantly upregulated in LSCs, with no effect on normal mouse hematopoiesis. Knocking down YTHDF1 inhibits tumorigenesis of both human and mouse AML cells in vivo and in vitro. Mechanistically, YTHDF1 promotes the translation of cyclin E2, thereby advancing AML progression. Interestingly, research has found that decitabine can inhibit YTHDF1, thereby suppressing YTHDF1‐regulated translation and leukemia development.^260^ Paris et al.^45^, ^238^ discovered that YTHDF2 inhibits TNF‐α expression through an m6A‐mediated mRNA decay mechanism, thereby suppressing apoptosis and promoting LSC self‐renewal as well as AML initiation and progression. Additionally, another study demonstrated that YTHDF2 is upregulated in t(8;21) AML and specifically targets AML1/ETO‐HIF1α.^261^ Inhibition of AML1/ETO or HIF1α downregulates YTHDF2 expression, enhancing methylation levels of TNFRSF1b mRNA and upregulating its expression, which promotes AML cell apoptosis and suppresses LSC proliferation.^261^ YTHDC1 has been reported to advance AML through different mechanisms. Analysis of CRISPR Screen data in AML cell lines revealed that the nuclear m6A reader protein YTHDC1 is crucial for AML cell survival. YTHDC1 is significantly overexpressed in various AML subtypes. Knockdown of YTHDC1 in AML cell lines or via CRISPR knockout slows AML cell proliferation, enhances myeloid differentiation, and increases apoptosis. In AML cell line and patient‐derived xenograft mouse models, YTHDC1 knockdown significantly inhibits AML development, further proving its critical role in AML progression.^117^ Mechanistic studies show that YTHDC1 forms compartmentalized structures in the nucleus, binding to m6A‐modified RNA and forming liquid‐like condensates known as nuclear YTHDC1–m6A condensates (nYACs). This process regulates downstream target gene MYC expression, and MYC overexpression can partially compensate for the phenotype induced by YTHDC1 knockdown.^117^ Unlike cytoplasmic m6A reader YTHDF2, which promotes m6A‐modified mRNA degradation, YTHDC1 binds m6A–mRNA to form nYACs, stabilizing its target mRNA and thus enhancing gene expression. Additionally, our laboratory found that YTHDC1 is significantly overexpressed in various karyotypic AML types. Knockdown of YTHDC1 significantly inhibits AML cell growth, increases apoptosis and differentiation. Using Ythdc1 knockout mouse models, we found that Ythdc1 knockout significantly suppresses AML cell growth in mice and impairs LSC function, extending the survival of AML mice. Mechanistic studies reveal that YTHDC1 stabilizes MCM4 mRNA by recognizing m6A modifications on MCM4.^241^ Re‐expression of MCM4 can significantly restore the growth suppression and increased apoptosis and differentiation caused by YTHDC1 loss. Another recent study reported that YTHDC1 interacts with RBM15, recruiting the PRC2 to RBFOX2 binding sites, thereby achieving chromatin silencing and transcriptional repression.^262^ The RBFOX2/m6A/RBM15/YTHDC1/PRC2 axis plays a critical role in myeloid leukemia. Downregulation of RBFOX2 significantly inhibits AML cell survival/proliferation and promotes myeloid differentiation. RBFOX2 is also essential for the self‐renewal of LSCs/LICs and the maintenance of AML.^262^

Another m6A reader protein, IGF2BP2, is also involved in regulating LSCs and AML progression. IGF2BP2 enhances the stability of MYC, SLC1A5, and GPT2 mRNA in an m6A‐dependent manner, promoting their translation and maintaining LSC/LIC function, thus accelerating AML progression.^263^ A recent interesting study found that IGF2BP2 is highly expressed in AML patient samples and is associated with poor prognosis. IGF2BP2 deficiency promotes apoptosis, damages LSC function, and inhibits AML development. Notably, the research team identified protein arginine methyltransferase PRMT6 as a potential key gene in leukemia initiation. They confirmed that IGF2BP2 regulates PRMT6 mRNA stability, making PRMT6 a downstream functional target of IGF2BP2.^264^ PRMT6 is highly expressed in AML samples and maintains LSC function; PRMT6 knockout inhibits AML development. The study further confirmed that the PRMT6 inhibitor EPZ020411 effectively suppresses LSC function.^264^ Research has shown that PRMT6 mainly catalyzes the formation of the repressive mark H3R2me2a. Upregulating PRMT6 downstream key target gene lipid transporter MFSD2A regulates the transport of fatty acids such as docosahexaenoic acid (DHA), maintaining LSC function.^264^ Additionally, Y box‐binding protein 1 (YBX1) regulates AML at the posttranscriptional level through interaction with IGF2BP2. Feng et al.^48^, ^115^ confirmed that YBX1 interacts with IGF2BP2, enhancing MYC and BCL‐2 mRNA stability and upregulating their translation in an m6A‐dependent manner, thereby promoting AML cell survival. This provides a theoretical basis for targeting YBX1 in AML treatment. Currently, the fundamental cause of AML resistance and relapse is the inability of chemotherapeutic agents to effectively eliminate LSCs. Both YTHDF2 and IGF2BP2 maintain LSC function and survival through m6A methylation modifications. Therefore, YTHDF2 and IGF2BP2 could serve as valuable targets for clearing LSCs, offering new possibilities for overcoming resistance and curing AML.

IGF2BP3 has been reported to be specifically overexpressed in AML and is essential for AML cell survival. Knocking down IGF2BP3 significantly inhibits cell apoptosis, reduces proliferation, and weakens leukemia ability of AML cells in vitro and in vivo. Mechanistic studies reveal that IGF2BP3 interacts with RCC2 mRNA and stabilizes the expression of m6A‐modified RNA.^265^ Furthermore, a recent studies have identified significant upregulation of the m6A reader proteins IGF2BP2 and IGF2BP3 in AML, regulated by super‐enhancers. The BRD4 inhibitor JQ1 has been shown to effectively inhibit the expression of IGF2BP2 and IGF2BP3. As target genes of IGF2BP2 and IGF2BP3, DDX21 significantly promotes AML cell proliferation, suppresses apoptosis, and induces cell cycle arrest.^266^ Additionally, it reduces tumor burden in mouse models and extends survival time. Mechanistic studies reveal that DDX21 interacts with the transcription factor YBX1 to enhance ULK1 transcription, thereby promoting AML progression.^266^ Similarly, research has reported upregulation of IGF2BP3 in high‐risk AML patients, with a positive correlation to SENP1 expression. IGF2BP3 binds to the SENP1 3′‐UTR in an m6A‐dependent manner, enhancing SENP1 expression and activating the AKT signaling pathway, which contributes to AML malignancy.^267^ IGF2BP3 is also known to directly bind EPOR mRNA, regulating its stability in an m6A‐dependent manner to facilitate AML progression^268^ (Figure 6). Overall, the above studies indicate that m6A readers protein play a crucial role in the progression of AML.

#### 6.1.4 m6A influence on chronic myeloid leukemia cells

**FIGURE 7:**
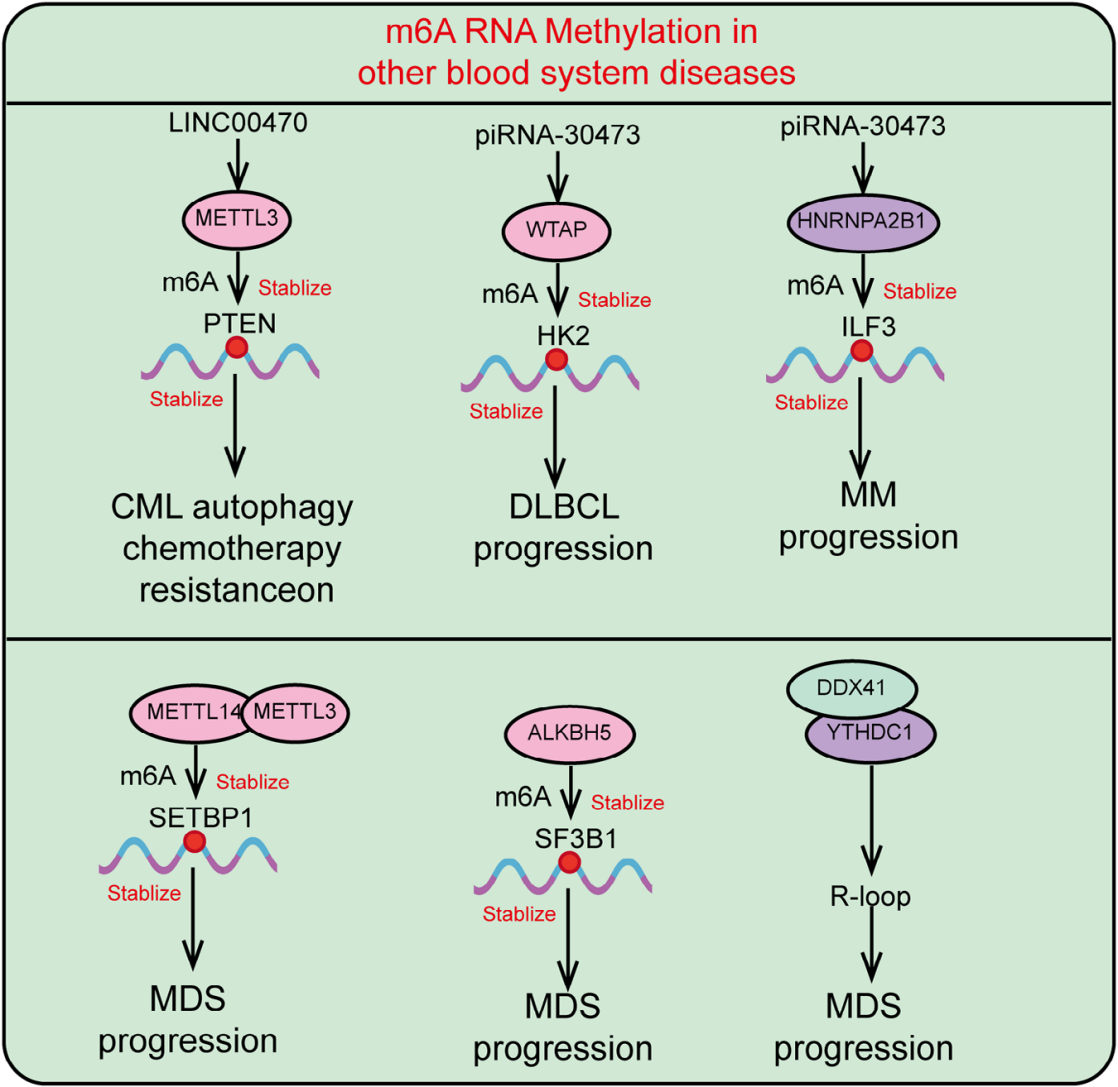
Mechanisms of m6A modification in the progression of other hematological malignancies. m6A methyltransferases, demethylases, and reader proteins contribute to the progression of hematologic malignancies by regulating RNA stability, degradation, and protein translation efficiency. PTEN, phosphatase and tensin homolog; HK2, hexokinase 2; ILF3, interleukin enhancer‐binding factor 3; SETBP1, SET binding protein 1; SF3B1, splicing factor 3b subunit 1; R‐LOOP, R‐loop refers to a three‐stranded nucleic acid structure (though not a gene, R‐loops form during transcription when RNA hybridizes with the DNA template strand, leaving a single‐stranded loop of DNA).

Previous research has demonstrated that PTEN, through its phosphatase activity, inhibits the PI3K‐AKT‐mTOR pathway and acts as a tumor suppressor in various cancer cells.^269^ In CML, PTEN has been shown to suppress the self‐renewal and survival of LSCs) thus exhibiting tumor‐suppressive effects. Recent studies have found that METTL3 is significantly enriched in the long intergenic ncRNA 470 (LINC00470) transcripts.^270^ LINC00470 positively regulates the m6A modification level of PTEN mRNA by binding with METTL3, promoting PTEN mRNA degradation and downregulating PTEN protein expression. This leads to inhibition of autophagy in CML cells and contributes to chemotherapy resistance (Figure 7). Overall, the above studies indicate that m6A demethylases play a crucial role in the progression of chronic myeloid leukemia.

#### 6.1.5 m6A influence on diffuse large B‐cell lymphoma cells

Diffuse large B‐cell lymphoma (DLBCL) is the most common subtype of lymphoid malignancies, with over a hundred classifications and high heterogeneity. Zhang et al.^271^ discovered that WTAP levels are persistently upregulated in DLBCL tissues, promoting cell proliferation, inhibiting apoptosis, and affecting chemotherapy efficacy. This upregulation depends on the stability provided by heat shock protein Hsp90, suggesting that Hsp90 inhibitors should be considered in chemotherapy regimens for WTAP‐high DLBCL.^271^, ^272^ Additionally, WTAP can form a complex with BCL6 (a transcriptional repressor blocking B‐cell differentiation), though the role of this complex in transcriptional activity or other functions requires further investigation. Literature reports also indicate that WTAP, as a specific target of piRNA‐30473 (a Piwi‐interacting RNA), is involved in regulating DLBCL cell proliferation and the cell cycle, closely related to patient prognosis. The mechanism involves piRNA‐30473 upregulating WTAP levels, which enhances the methylation and expression of the key target gene HK2 mRNA, thereby promoting DLBCL cell survival and proliferation^273^ (Figure 7). Overall, the above studies indicate that m6A demethylases play a crucial role in the progression of DLBCL.

#### 6.1.6 m6A influence on multiple myeloma cells

Jiang et al. demonstrated through cell proliferation assays, in vitro knockouts, and subcutaneous tumorigenesis experiments that HNRNPA2B1 promotes multiple myeloma (MM) cell proliferation and inhibits apoptosis in both in vivo and in vitro settings. Silencing HNRNPA2B1 reduces ILF3 m6A modification and transcription. Knocking down ILF3 reverses HNRNPA2B1‐induced cell proliferation, indicating that ILF3 is a crucial m6A/HNRNPA2B1 target in MM cells.^274^ Further analysis showed that AKT3 mRNA is enriched at ILF3 binding sites, with its expression positively regulated by HNRNPA2B1. This study highlights that HNRNPA2B1 mediates increased AKT3 expression by enhancing m6A‐dependent ILF3 mRNA stability, thus playing a critical role in MM progression and correlating with poor prognosis^274^ (Figure 7). Overall, the above studies indicate that m6A demethylases play a crucial role in the progression of multiple myeloma.

#### 6.1.7 m6A modification in myelodysplastic syndromes

Research indicates that RNA m6A epigenetic dysregulation exists in the bone marrow of myelodysplastic syndromes (MDS) patients, with elevated m6A modification levels closely associated with high‐risk MDS. METTL14 is a key regulator causing m6A modification disorders in MDS RNA. Silencing METTL14 significantly reduces RNA m6A levels in MDS cells, markedly suppresses cell proliferation and colony formation, and prolongs mouse survival. Mechanistic studies reveal that METTL14 enhances SETBP1 mRNA m6A modification by forming a heterodimer with METTL3, increasing SETBP1 mRNA stability, and activating the PI3K‐AKT signaling pathway, thereby promoting MDS development.^275^ ALKBH5 is reported to regulate SF3B1 5′‐UTR m6A demethylation, affecting SF3B1 translation, DNA repair, and epigenetic regulator splicing, driving MDS progression.^276^ Studies also show that DDX41 promotes YTHDC1 recruitment to the R loop by enhancing METTL3 and YTHDC1 binding, maintaining genomic stability in MDS and promoting disease progression^277^ (Figure 7). Overall, the above studies indicate that m6A demethylases play a crucial role in the progression of MDS.

### 6.2 The role of M7G modification in hematologic malignancies

Recent studies have shown that METTL1 and WDR4 are significantly upregulated in patients with acute AML, and their expression levels are closely associated with poor prognosis in these patients. Knockdown of METTL1 significantly inhibits AML cell proliferation, promotes apoptosis, and causes G1 phase cell cycle arrest, thereby suppressing the progression of AML cells in vitro. Mechanistic studies suggest that METTL1 may specifically mediate the m7G modification of tRNA, regulating the stability and biogenesis of tRNA carrying the m7G modification. This, in turn, affects the translation efficiency of proteins involved in translation regulation processes and enzyme activity within the cell.^278^ The m7G modification plays a critical role in the progression of AML by regulating RNA metabolism and protein translation.

### 6.3 The role of M5C modification in hematologic malignancies

In leukemia, NSUN1 specifically interacts with BRD4 and directly binds to the CTD‐S2P of RNA polymerase II (RNA‐pol II). In 5‐azacytidine (5‐AZA)‐resistant leukemia cells, a unique NSUN1/BRD4/RNA‐pol II CTD‐S2P complex is formed, mediating the development of 5‐AZA‐resistant chromatin structures and contributing to 5‐AZA resistance in leukemia. Conversely, NSUN3 and DNMT2 exhibit opposing effects on 5‐AZA‐sensitive leukemia cells. Mechanistically, the RNA‐binding protein hnRNPK directly interacts with m5C methyltransferases NSUN3 and DNMT2, lineage‐determining transcription factors GATA1 and SPI1/PU.1, and CDK9/PTEFb, forming a unique complex at nascent RNA sites, which ultimately results in a 5‐AZA‐sensitive chromatin structure.^279^ Comparative analysis of bone marrow samples from 5‐AZA‐resistant and ‐sensitive leukemia patients reveals significantly higher levels of m5C mRNA in the 5‐AZA‐resistant samples. The expression levels of hnRNPK, NSUN1, and BRD4 are associated with leukemia progression and contribute to 5‐AZA resistance and tumor development.^279^ Research reports indicate that YBX1 maintains the survival of myeloid leukemia cells in an m6A‐dependent manner, while having no effect on normal hematopoiesis. YBX1 interacts with m6A readers IGF2BPs through its conserved cold shock domain to indirectly bind m6A‐modified mRNA, thereby enhancing the stability of apoptosis‐related genes MYC and BCL2, which in turn sustains the function of leukemia cells.^115^ Recent research indicates that TET2 regulates the accumulation of m5C modifications in TSPAN13 mRNA. These m5C modifications are specifically recognized by YBX1, which increases the stability and expression of TSPAN13 transcripts. This process promotes leukemia development, LSC migration/homing, and LSC self‐renewal.^127^ Recently, a notable study identified another novel RNA m5C methylation binding protein, SRSF2, and revealed its association with leukemia development. Further research found that the SRSF2P95H mutation in leukemia inhibits SRSF2 recognition of m5C, affecting mRNA alternative splicing mediated by SRSF2, and leukemia patients with impaired SRSF2‐m5C binding have poor prognosis.^280^ YBX2 has recently been reported as a novel mammalian m5C‐binding protein capable of undergoing liquid–liquid phase separation both in vivo and in vitro.^281^, ^282^ Other methylation binding proteins remain to be discovered and validated, and their regulatory mechanisms on RNA m5C modifications require further investigation. The m5C modification plays a critical role in the progression of AML by regulating RNA metabolism and protein translation.

### 6.4 The role of ac4C modification in hematologic malignancies

As a hematological malignancy, AML patients have higher NAT10 expression. Compared with patients with low NAT10 expression, those with high NAT10 expression in AML have significantly shorter survival times.^283^ Knocking down NAT10 promotes apoptosis and differentiation in AML cells, inhibits cell proliferation, and induces cell cycle arrest. Additionally, NAT10 regulates the expression of cell cycle proteins and upregulates p21 expression to control the AML cell cycle. NAT10 activates the classical apoptosis pathway through Bax/Bak and downregulation of Bcl‐2, promoting AML progression.^284^ Another similar study indicated that NAT10 expression is higher in NPM1 mutant patients than in NPM1‐wt patients and is negatively correlated with progression‐free survival and overall survival.^283^ The ac4C modification plays a critical role in the progression of AML by regulating RNA metabolism and protein translation.

### 6.5 Crosstalk between histone modifications and m6A modifications in AML

Recent studies have revealed significant upregulation of IGF2BP2 and PRMT6 in AML. Knocking down or deleting IGF2BP2 and PRMT6 impairs AML progression and LSC function. Mechanistically, IGF2BP2 stabilizes PRMT6 mRNA via m6A modification. As a classical protein arginine methyltransferase, PRMT6 mediates H3R2me2a methylation, which suppresses the expression of the lipid transporter MFSD2A, leading to increased levels of DHA and impairing the self‐renewal capacity of LSCs.^264^ Additionally, another study from the same group found that the epigenetic enzyme KDM4C reduces H3K9me3 levels at the ALKBH5 promoter, increasing chromatin accessibility at the ALKBH5 gene locus. This recruits MYB and RNA‐pol II, upregulating ALKBH5 expression. ALKBH5, in turn, affects the mRNA stability of receptor tyrosine kinase AXL through an m6A‐dependent mechanism, promoting LSC self‐renewal and AML progression, while ALKBH5 has no impact on normal hematopoiesis.^258^ Furthermore, the deubiquitinase USP9X removes ubiquitination from ALKBH5 methyltransferase, maintaining its protein stability and promoting LSC self‐renewal and AML progression.^285^ Another interesting study found that HIF1α is abnormally overexpressed in AML, functioning as an oncogene by activating DNMT 3α, leading to DNA hypermethylation and promoting the expression of WTAP, a key component of the m6A MTC, thereby driving AML progression. Mechanistically, HIF1α directly binds to the hypoxia response element of the WTAP promoter, activating WTAP gene expression. This increases m6A levels on KDM4B transcripts, promoting their degradation, reducing KDM4B expression, and increasing H3K9 trimethylation, thereby inhibiting AML cell growth both in vitro and in vivo.^286^ SENP1 has also been reported to be overexpressed in AML and is associated with shorter patient survival. SENP1 expression is stabilized by IGF2BP3 in an m6A‐dependent manner, and SENP1 promotes AML cell proliferation and inhibits apoptosis by regulating the AKT/mTOR signaling pathway. Furthermore, SENP1 desumoylates HDAC2, enhancing EGFR transcription and activating the AKT pathway.^267^ Overall, these studies reveal the critical role of the crosstalk between histone posttranslational modifications and m6A modifications in regulating LSC self‐renewal and AML progression.

### 6.6 Crosstalk between m5C and m6A modifications in AML

Chen and colleagues^127^ found that TET2 is abnormally upregulated in AML, and its deficiency promotes LSC self‐renewal. Mechanistic studies revealed that TET2 deficiency leads to elevated m5C modification levels on TSPAN13 mRNA in an m5C‐dependent manner. This modification is specifically recognized by YBX1, which stabilizes TSPAN13 transcripts and activates the CXCR4/CXCL12 signaling pathway, enhancing LSC homing and migration to the bone marrow microenvironment.^127^ In summary, these studies highlight the critical role of the crosstalk between m5C and m6A modifications in regulating LSC self‐renewal and AML progression.

### 6.7 Crosstalk between superenhancers and m6A modifications in AML

Superenhancers have recently been reported to promote AML progression and LSC self‐renewal. One study showed that superenhancer‐driven H3K27ac, H3K4me1, and BRD4 significantly upregulate IGF2BP2 and IGF2BP3 in AML, stabilizing DDX21 mRNA in an m6A‐dependent manner. DDX21 is also overexpressed in AML and interacts with the m5C recognition protein YBX1 to transcriptionally activate ULK1 expression. This promotes AML cell proliferation, apoptosis, and cell cycle arrest.^266^ In conclusion, this study reveals the critical role of the crosstalk between superenhancers and m6A modifications in AML progression. In conclusion,, these various RNA modifications, along with their interactions with other epigenetic modifications, play crucial roles in the progression of malignant hematological diseases, particularly AML.

## 7 TARGETING SIGNALING PATHWAYS AND M6A MODIFICATIONS IN LEUKEMIA TREATMENT

Therapeutic strategies targeting signaling pathways and m6A modifications have shown significant potential in leukemia research. Signaling pathways such as Wnt/β‐catenin, NF‐κB, and Hedgehog play crucial roles in the proliferation, apoptosis, and differentiation of leukemia cells. By targeting these aberrantly activated pathways, the growth of leukemia cells can be effectively inhibited, promoting their apoptosis. At the same time, m6A modification, an essential epigenetic regulatory mechanism, modulates gene expression in leukemia cells by affecting mRNA stability, splicing, and translation. Key enzymes involved in m6A modification, including METTL3, FTO, and ALKBH5, have been shown to play critical roles in the initiation and progression of leukemia. Targeting these enzymes or the regulatory mechanisms of m6A modification presents novel therapeutic opportunities to suppress the malignant properties of leukemia cells and improve patient outcomes. Therefore, the combination of signaling pathway inhibitors and m6A modification regulators may represent a promising direction for future leukemia treatment.

### 7.1 Summary of targeted signal pathway inhibition for leukemia treatment

Signal pathways associated with LSCs play varying roles in regulating their self‐renewal, proliferation, differentiation, and apoptosis.^287^ Targeting and inhibiting these pathways theoretically aims to eliminate LSCs and thus treat leukemia more effectively.

#### 7.1.1 Targeting the Wnt/β‐catenin signaling pathway

Indomethacin, a reversible cyclooxygenase inhibitor, blocks the activation of the Wnt pathway by inhibiting β‐catenin expression. Studies by Wang et al.^37^ have shown that indomethacin treatment can suppress LICs crucial for AML development and affect established LSCs.^288^ In addition to single‐agent inhibitors, combination therapy has also shown promising results in targeting LSCs. Lin et al.^289^ assessed the effects of sulfadiazine (SFN) on Imatinib (IM)‐resistant LSCs and found that combining SFN with IM effectively eradicates CD34+/CD38− LSCs in CML. The efficacy of SFN in targeting LSCs and reducing resistance primarily involves downregulation of β‐catenin expression.^289^ At low doses, doxorubicin (DXR) acts as an inhibitor of Akt–β‐catenin interactions.^290^ The synergistic effect of Wnt/β‐catenin and PI3K/AKT pathways can promote tumorigenesis and resistance to therapy. Results by Perry et al.^290^ indicated that combined chemotherapy and low‐dose DXR significantly improved survival in leukemia model mice, with low‐dose DXR exhibiting stronger specificity for targeting LSCs, including those resistant to chemotherapy.

#### 7.1.2 Targeting the Hedgehog signaling pathway

Glasdegib, a selective small‐molecule inhibitor of SMO, has demonstrated efficacy against AML cells both in vitro and in vivo using human xenograft mouse models.^291^ Its shorter half‐life helps reduce toxicity to normal stem cells and enhance its ability to target LSCs, particularly when used in combination with other agents. The United States Food and Drug Administration has approved Glasdegib in combination with low‐dose Ara‐C for newly diagnosed elderly AML patients (≥75 years old) or those who cannot tolerate intensive induction therapy and have significant comorbidities.^291^ Cyclopamine, a natural plant steroidal alkaloid and SMO inhibitor, has been shown to reduce the resistance of CD34+ leukemia cell lines to cytarabine and DXR.^292^

#### 7.1.3 Targeting the NF‐κB signaling pathway

Parthenolide (PTL) is an NF‐κB kinase activation inhibitor that can inhibit LSC proliferation and induce G2 phase cell cycle arrest. PTL is the first effective natural drug targeting LSCs, meaning it preferentially kills leukemia stem/progenitor cells with minimal impact on noncancer cells.^293^ PTL treatment significantly reduces the engraftment potential of AML patient‐derived LSCs, but its poor druggability limits clinical application. Ghantous et al.^42^, ^294^ identified a new compound, dimethylamino parthenolide, which, as an NF‐κB inhibitor, exhibits similar activity to PTL and selectively eradicates LSCs. DMAMCL (ACT001) also eliminates LSCs by inhibiting NF‐κB activity.^294^ Regulating aberrant signaling pathways in AML through these inhibitors can effectively target and clear LSCs, thus improving AML prognosis. Therefore, targeted inhibition of signaling pathways remains a promising direction for future research.

#### 7.1.4 Potential challenges and limitations of targeted signaling pathway therapies

Targeted signaling pathway therapies for leukemia have made significant advances in recent years, yet they continue to face several potential challenges and limitations. First, the heterogeneity of leukemia is considerable, with tumor cells from different patients potentially exhibiting distinct signaling pathway abnormalities. This heterogeneity implies that a single targeted therapy may not be uniformly effective across all patients, necessitating personalized treatment approaches to address the specific signaling abnormalities present in individual cases. Second, targeted signaling pathway therapies may induce compensatory mechanisms or activate alternative signaling pathways within tumor cells, potentially reducing therapeutic efficacy or leading to resistance. For instance, drugs targeting a specific signaling pathway might enable cancer cells to continue growth and survival through other pathways. Additionally, targeted therapies may have off‐target effects on normal cells, as some signaling pathways are also crucial for normal cellular functions. Therefore, improving the specificity and selectivity of these drugs is essential to minimize adverse effects on healthy tissues. Finally, the long‐term efficacy and safety of targeted signaling pathway therapies require further investigation to assess their impact on patients' quality of life and potential long‐term toxicities. In conclusion, optimizing the effectiveness of targeted signaling pathway therapies for leukemia involves addressing these challenges and continually refining treatment strategies.

### 7.2 Summary of targeting m6A modification in leukemia treatment

**FIGURE 8:**
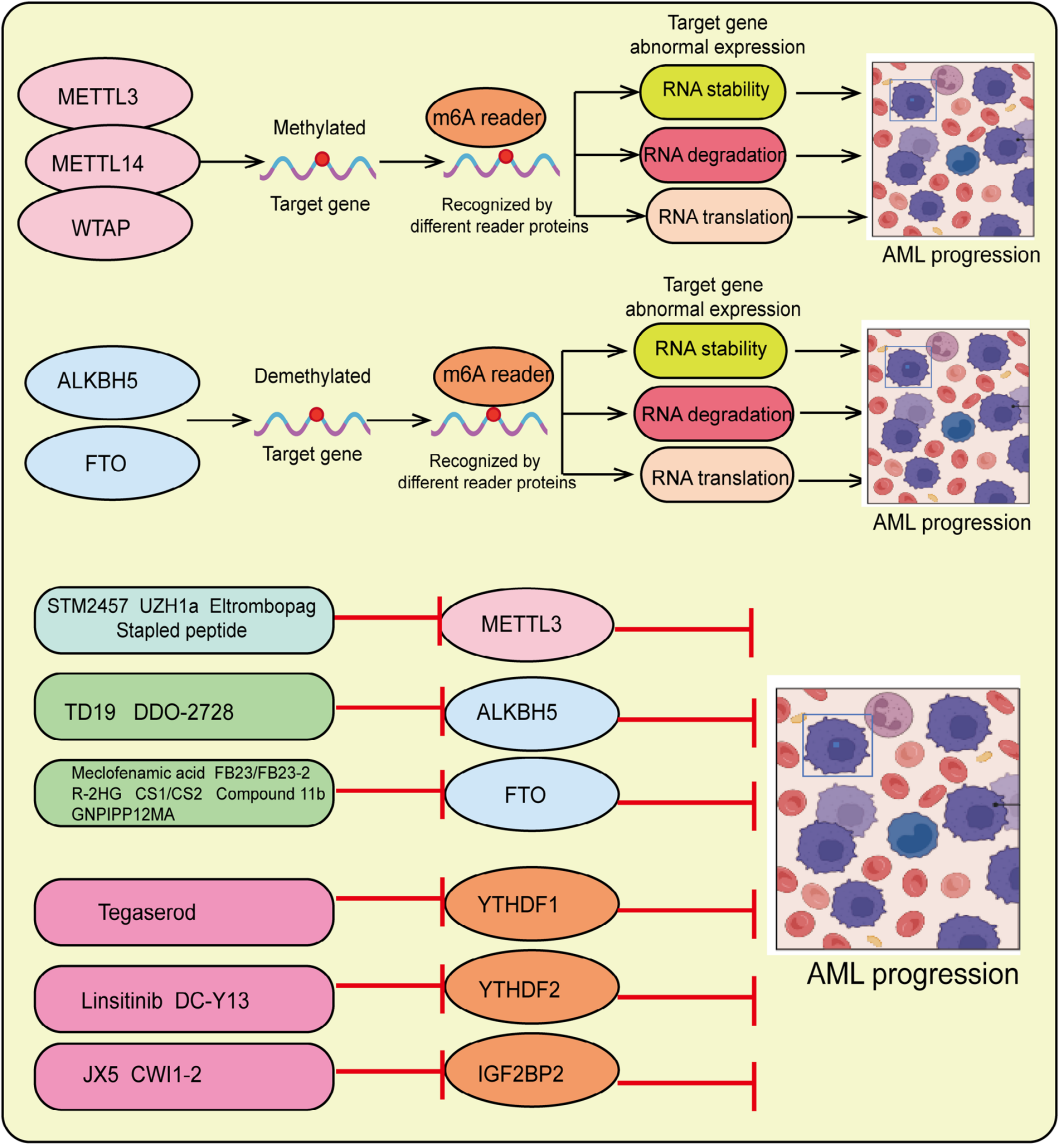
The role and mechanisms of m6A modifications in the progression of AML, and targeting m6A regulatory factors to inhibit leukemia progression.

M6A methylation modification, the most prevalent form of RNA methylation, plays a role in RNA metabolism, processing, and gene transcription. It links ncRNAs and histone modifications to the occurrence and development of AML, thereby regulating AML cell proliferation, differentiation, apoptosis, and the self‐renewal of LSCs.^295^ As m6A modification is closely related to the pathogenesis and drug resistance of AML, m6A‐related components represent potential targets for AML treatment. With advancements in high‐throughput sequencing technologies, a growing number of m6A‐related inhibitors have been developed, providing more therapeutic options for AML.^248^ Based on the different types of targeted m6A modifications, small‐molecule modulators can be classified into three categories: modulators targeting writers, erasers, and readers. Among these, the majority of small molecules target writers and erasers. Below, we provide an overview of small molecules targeting m6A modifications, focusing on their activity and mechanisms of action (Figure 8 and Table 1).

**TABLE 1:** Summary of m6A‐related inhibitors.

Regulator	Inhibitors	Mechanism	References
METTL3	STM2457	Binding to the S‐adenosylmethionine site decreases the expression of BRD4, SP1, and c‐Myc	^248^
METTL3	UZH1a	Reduces METTL3 activity	^296^
METTL3	Eltrombopag	Binds to the METTL3‐14 complex to block the methyltransferase activity of the METTL3 subunit	^298^
METTL3	Stapled peptide	Reduces METTL3 activity	^308^
ALKBH5	TD19	Binds to ALKBH5 residues C100 and C26, inhibiting the demethylase activity of ALKBH5	^304^
ALKBH5	DDO‐2728	Reduces the stability of TACC3 mRNA, promoting AML cell apoptosis	^305^
FTO	Meclofenamic acid	Reduces FTO activity	^309^
FTO	FB23/FB23‐2	Inhibits FTO's m6A demethylation, downregulates MYC and CEBPA expression	^256^
FTO	FB23/FB23‐2	increases RARA and ASB2 protein levels, thereby inhibiting AML cell proliferation	^255^
FTO	R‐2HG	Competitively inhibits FTO, reducing MYC and CEBPA protein levels	^300^
FTO	CS1/CS2	reducing MYC and CEBPA protein levels; inhibits LILRB4 expression, activating T lymphocyte toxicity	^256^
FTO	Compound 11b	Inhibits FTO's m6A demethylation, downregulates MYC, and upregulates RARA protein levels	^302^
FTO	GNPIPP12MA	Promotes m6A RNA modification, reducing LSCs transcription	^303^
YTHDF1	Tegaserod	Inhibits YTHDF1 expression and its binding to RNA	^260^
YTHDF2	Linsitinib	Inhibits YTHDF2 activity and expression	^306^
YTHDF2	DC‐Y13	Inhibits YTHDF2 binding to RNA	^310^
IGF2BP2	JX5	Inhibits IGF2BP2 activity and expression levels	^307^
IGF2BP2	CWI1‐2	Directly binds to IGF2BP2, competitively inhibiting its m6A binding protein activity	^263^

Abbreviation: Eltrombopag, Eltrombopag olamine.

#### 7.2.1 Inhibitors of m6A methylation methyltransferases

Yankova et al.^248^ identified a METTL3 inhibitor, STM2457, which reduces the methyltransferase activity of METTL3 by binding to the SAM binding site. This leads to decreased m6A methylation of METTL3 target genes such as BRD4, SP1, and c‐Myc, thereby inhibiting their translation and inducing apoptosis while suppressing AML cell proliferation.^248^ UZH1a, a selective, cell‐permeable non‐nucleoside METTL3 inhibitor, has a stereoisomer UZH1b. Takayuki Murata et al.^296^ found that increasing doses of UZH1a decreased m6A methylation levels of mRNA transcripts in MOLM‐13 cells in a dose‐dependent manner and downregulated METTL3 activity, thus promoting leukemia cell apoptosis and inhibiting disease progression. Additionally, Ding et al.^60^ discovered that eltrombopag could block the methyltransferase activity of METTL3 by binding to the METTL3 subunit in the METTL3‐14 complex. This results in reduced m6A methylation of target gene mRNAs, promoting AML cell apoptosis and inhibiting cell proliferation. The study also found that eltrombopag has a synergistic effect with other leukemia treatments such as venetoclax and azacitidine, delaying leukemia progression.^297^, ^298^

#### 7.2.2 Inhibitors of m6A demethylases

The nonsteroidal anti‐inflammatory drug meclofenamic acid (MA) inhibits FTO activity. Huang et al.^299^ optimized MA to produce two derivatives, FB23 and FB23‐2, which specifically inhibit FTO‐mediated m6A demethylation. This results in increased m6A levels in MYC, CEBPA, RARA, and ASB2 mRNA transcripts, downregulation of MYC and CEBPA proteins, and upregulation of RARA and ASB2 proteins, thereby inhibiting AML cell proliferation.^299^ R‐2HG, an FTO inhibitor produced by isocitrate dehydrogenase 1/2 (IDH1/2), also inhibits FTO competitively, promotes m6A methylation, decreases the stability of MYC and CEBPA mRNAs, and suppresses AML cell proliferation. Moreover, R‐2HG enhances AML cell sensitivity to low‐methylation agents such as azacitidine and decitabine, promoting AML cell apoptosis and helping prevent HMA‐induced drug resistance.^300^ Another study developed two FTO inhibitors, CS1 and CS2, which inhibit LILRB4 expression and activate T lymphocyte toxicity. CS1 and CS2 bind directly to FTO residues such as H231, E234, K216, S229, and H231, inhibiting its methyltransferase activity and increasing the m6A abundance of MYC, CEBPA, and snRNA mRNAs, thereby inhibiting leukemia progression.^301^ Subsequently, Prakash et al.^302^ designed compound 11b, which increases m6A methylation levels in FTO, downregulates MYC, and upregulates RARA protein levels, thereby inhibiting AML cell survival. This suggests the potential for new synthetic inhibitors in clinical applications.^302^ A team designed the FTO inhibitor GNPIPP12MA, which increases m6A mRNA modification and reduces LSC expression, targeting LSCs and leukemia cells.^303^ Lai et al.^304^ discovered a new selective ALKBH5 inhibitor, TD19, which selectively binds to ALKBH5 residues C100 and C26. By inhibiting ALKBH5's binding to target mRNA, TD19 downregulates ALKBH5's m6A demethylase activity, exerting antileukemia effects.^304^ Additionally, Wang et al.^55^, ^305^ developed the ALKBH5 selective inhibitor DDO‐2728, which increases m6A abundance on TACC3 mRNA transcripts, decreases mRNA stability, and promotes AML cell apoptosis. DDO‐2728 significantly inhibited the growth of primary cells in xenografted mice, showing antileukemia effects. Both TD19 and DDO‐2728, as newly developed targeted agents, offer high selectivity and efficacy compared with dual FTO/ALKBH5 inhibitors like IOX3, MV1035, ALK‐04, and cmp‐3. They selectively target ALKBH5 without affecting FTO, providing more specific regulation of AML cells and promoting tumor cell apoptosis.

#### 7.2.3 Inhibitors of m6A reader proteins

Inhibitors of m6A reader proteins play significant roles in the treatment of various malignancies. YTHDF1 is dispensable for normal hematopoiesis but significantly promotes AML progression. Molecular docking studies have revealed that tegaserod can specifically and concentration‐dependently bind directly to the YTH domain of YTHDF1. This binding blocks the interaction between YTHDF1 and m6A‐modified target genes, inhibiting the translation of downstream target genes and suppressing AML progression.^260^ Dixit et al.^306^ studied Linsitinib in glioblastoma, an IGF1/IGF1R inhibitor, and found that it reduces glioblastoma stem cell activity by downregulating YTHDF2 protein levels, thereby inhibiting tumor cell proliferation and slowing disease progression. Wang et al.^310^ found that the YTHDF2 inhibitor, DC‐Y13, directly inhibits the binding of YTHDF2 to m6A‐containing RNA, thereby suppressing tumor growth. More interestingly, DC‐Y13‐27 can further enhance the effects of radiotherapy and anti‐PD‐L1 therapy. The combination of DC‐Y13‐27 and anti‐PD‐L1 significantly slowed the growth of MC38 tumors, while the triple therapy of DC‐Y13‐27, IR, and anti‐PD‐L1 produced the strongest antitumor effects.^117^ Feng et al.^307^ developed a small‐molecule IGF2BP2 inhibitor, JX5, for ALL, which lowers m6A methylation levels by inhibiting IGF2BP2. JX5 weakens the stability of NOTCH1 mRNA, thereby downregulating its expression and inhibiting leukemia cell proliferation.^307^ In AML research, Weng et al.^263^ designed a small‐molecule IGF2BP2 inhibitor, CWI1‐2, which binds directly to IGF2BP2 and competitively inhibits its m6A‐binding protein activity. This suppresses AML cell proliferation and shows significant antileukemia effects. The study also found that CWI1‐2 in combination with other leukemia chemotherapy drugs, such as DXR and vincristine, exhibits good therapeutic effects in AML treatment.^263^ However, there is limited research on the application of reader protein inhibitors in AML treatment, suggesting that m6A reader proteins may have more complex mechanisms in hematologic malignancies, particularly AML, which warrants further investigation. Overall, the use of m6A‐related inhibitors, either alone or in combination with chemotherapy, demonstrates significant anti‐AML potential and lays the groundwork for the development of new targeted drugs.

#### 7.2.4 Clinical significance of m6A‐targeted therapies

Targeted m6A therapies have demonstrated significant potential in the treatment of leukemia. m6A modification is a prevalent epigenetic alteration found on RNA that plays a crucial role in regulating gene expression, cellular differentiation, and tumorigenesis. In leukemia cells, aberrant m6A modifications are often closely associated with cancer development and progression. Therefore, targeted m6A therapies have the potential to improve leukemia treatment outcomes by modulating these modifications. First, targeting m6A can regulate leukemia cell proliferation and apoptosis by inhibiting or activating specific m6A methyltransferases (e.g., METTL3) or demethylases (e.g., FTO). By altering m6A modification levels, these therapies can correct abnormal transcription and translation processes in leukemia cells, thereby suppressing tumor growth. Second, m6A modifications have profound effects on posttranscriptional regulation; thus, m6A‐targeted drugs may enhance therapeutic efficacy by influencing signaling pathways and transcription factors in leukemia cells. For example, modulating m6A modifications could improve cellular sensitivity to chemotherapeutic agents, reduce drug resistance, and consequently enhance treatment effectiveness. Finally, combining m6A‐targeted therapies with other treatment strategies (e.g., targeted drug therapy, immunotherapy) may further enhance the overall efficacy of leukemia treatment. By integrating various therapeutic approaches, a more comprehensive attack on leukemia can be achieved, potentially improving patient survival rates and quality of life. In conclusion, m6A‐targeted therapies offer new hope for leukemia patients, and future research will focus on optimizing these strategies and evaluating their practical applications in clinical settings.

#### 7.2.5 Potential challenges and limitations of m6A‐targeted therapies

m6A‐targeted therapies, as an emerging therapeutic strategy, hold significant promise but also encounter several challenges and limitations. First, the complexity and diversity of m6A modifications mean that our understanding of their mechanistic roles remains incomplete. m6A modifications not only affect RNA stability and translation but are also involved in various cellular processes, thus targeting specific m6A sites may inadvertently impact other normal biological functions. Second, the selectivity and specificity of regulatory factors involved in m6A modifications (such as methyltransferases and demethylases) require further investigation to minimize side effects and enhance therapeutic efficacy. Additionally, m6A‐targeted therapies may face challenges related to drug delivery and biodistribution, as these therapeutics need to precisely reach and act within target cells. Finally, genetic variability among individuals may influence therapeutic responses, with some patients potentially developing resistance, leading to variability in treatment efficacy. Therefore, a thorough exploration of these potential challenges is essential before advancing m6A‐targeted therapies, and appropriate strategies must be developed to improve their clinical safety and effectiveness.

## 8 CONCLUSION AND PROSPECTS

HSCs are located at the apex of the hematopoietic hierarchy and possess the ability to differentiate into various progenitors and blood cells. HSCs maintain hematopoietic system homeostasis through the regulation of self‐renewal and multilineage differentiation. Disruption of this balance can lead to a range of hematological malignancies, such as leukemia, aplastic anemia, and MDS. Therefore, understanding the underlying mechanisms that regulate HSC homeostasis is of great significance. The homeostasis of the hematopoietic system is finely regulated by lineage‐specific transcription factors, epigenetic regulators (including DNA methylation, histone modifications, and RNA methylation), and cellular metabolism. Among these, m6A is the most prevalent modification on eukaryotic mRNA, impacting various aspects of mRNA metabolism, including stability, degradation, localization, and splicing. As a key form of RNA epigenetic regulation, m6A modification has garnered widespread attention due to its role in both normal hematopoiesis and LSCs. m6A modifications regulate gene expression and signaling pathways by influencing RNA stability, translation efficiency, and splicing, thereby playing a critical role in cell growth, differentiation, and self‐renewal. In normal hematopoiesis, m6A finely tunes HSC fate decisions to maintain hematopoietic balance. Studies have demonstrated that m6A modification regulates the expression of key genes, promoting HSC self‐renewal and multipotency while preventing excessive differentiation. However, aberrant m6A modifications in LSCs are closely associated with tumorigenesis and progression. Leukemia is a malignancy driven by LSCs, characterized by abnormal proliferation and blocked differentiation of LSCs. The dysregulated expression of m6A in leukemia promotes LSC survival and self‐renewal by influencing critical genes and signaling pathways. Targeting m6A modification with specific inhibitors presents a promising therapeutic strategy.^311^ For instance, inhibiting the activity of the m6A methyltransferase METTL3 reduces m6A levels, thereby suppressing leukemia cell proliferation and survival.^248^ Additionally, inhibitors targeting m6A demethylases, such as FTO and ALKBH5, have demonstrated potent antitumor activity.^299^ Regulating m6A levels to disrupt leukemia cell signaling and gene expression effectively inhibits tumor growth. Furthermore, signaling pathway inhibitors have shown great potential in leukemia treatment. Given that m6A modifications influence leukemia progression through multiple signaling pathways, combining signaling pathway inhibitors with m6A regulators could enhance therapeutic outcomes.^5^

m6A modifications play a critical role in various biological processes and tumorigenesis. Several oncogenic m6A modifications have been identified as promising targets for cancer therapy. Despite extensive research on the role of RNA m6A modifications in cancer and their underlying molecular mechanisms, many questions remain unresolved. For example, why do m6A methyltransferases (writers) and demethylases (erasers), as well as m6A‐binding proteins (readers), exhibit similar oncogenic or tumor‐suppressive roles in the same cancer type? In AML, writers (such as METTL3, METTL14, and METTL16), erasers (FTO and ALKBH5), and readers (YTHDF1, YTHDF2, YTHDC1, and IGF2BP2) are all aberrantly upregulated, promoting AML progression.^4^, ^28^ How these m6A‐modifying factors coordinate the epitranscriptomic regulation in specific cancer types, particularly at the single‐cell level, remains unclear. Additionally, the role of m6A modifications in cross‐talk between cancer cells, immune cells, and the tumor microenvironment (TME) requires further exploration.

Systematic studies addressing these questions will enhance our understanding of the role of m6A in cancer and provide a stronger foundation for developing m6A‐based therapeutic approaches. In the context of hematological malignancies, m6A modifications are at the forefront of research, playing crucial roles in tumorigenesis, progression, immune responses, and drug resistance. However, elucidating their precise role within the TME requires more cost‐effective sequencing methods and in‐depth molecular studies. Most current conclusions in the field are based on knockout studies of key components of the m6A modification complex, while mammalian transcriptomes contain thousands of methylation sites. Identifying causally related sites, distinguishing distinct molecular effects (such as those on stability, translation, or localization), and understanding their role in different phases of the mRNA life cycle remain challenges. Establishing connections between specific methylated mRNA, molecular mechanisms, and phenotypes represents a major challenge.

Small‐molecule inhibitors, PROTAC‐based inhibitors, transcriptome editing, and combination therapies hold strong therapeutic potential for patients with hematological malignancies.^312^ Further research on how and under what conditions RNA modifications control gene expression, the specific mechanisms of each m6A reader, and how they are embedded and regulated within cellular circuits will help elucidate the pathogenic mechanisms of m6A and enhance the precision of clinical targeting for hematological malignancies.

Research methods in the study of m6A modifications face various challenges and limitations. The difficulty in identifying specific methylated mRNA remains a major issue. The widespread and dynamic nature of m6A modifications complicates the accurate detection and quantification of methylation sites on specific mRNA. Existing techniques, such as m6A‐seq, while providing genome‐wide m6A modification maps, face issues of low resolution and high background noise, limiting the precise identification of target mRNA.^11^ Establishing a causal relationship between m6A modification and cellular phenotypes presents a significant challenge. m6A modifications play multifaceted roles in regulating gene expression and cellular functions, often relying on upstream and downstream signaling pathways. Therefore, determining how m6A specifically affects cellular phenotypes and its precise contribution to disease states remains a complex, unresolved issue. To address these challenges, future research must develop more efficient techniques to enhance the precision of m6A detection and utilize functional experiments to explore the specific relationship between m6A modifications and phenotypes.

Due to the limitations of traditional m6A techniques, such as high RNA input requirements and a lack of single‐nucleotide resolution and quantitative m6A data, a single‐nucleotide resolution m6A map from patient‐derived tumor samples has yet to be reported. However, with advanced m6A‐seq methods (e.g., m6A–SAC‐seq, GLORI, and eTAM‐seq),^313^, ^314^ it is feasible to generate quantitative transcriptome‐wide m6A maps at single‐nucleotide resolution from clinical samples. Such maps could enable the identification of m6A signatures from specific mRNA loci, potentially serving as biomarkers for cancer diagnosis, classification, prognosis, risk stratification, and treatment selection. Future research on m6A modification could focus on several key areas. First, developing more cost‐effective sequencing methods is crucial. Although current m6A sequencing techniques provide rich characterization data, their high cost and complex workflows limit broader application. Therefore, researchers should work on improving existing technologies to simplify the sequencing process and reduce experimental costs, enabling large‐scale, high‐throughput m6A modification detection. This would facilitate a more comprehensive understanding of the role of m6A modifications in various biological processes and disease states. Second, further development of single‐nucleotide resolution mapping techniques for ncRNA m6A modifications may lead to the identification of a new class of biomarkers. Moreover, improving high‐throughput m6A‐seq technology to allow quantitative, transcriptome‐wide, single‐nucleotide resolution analysis with minimal RNA input from numerous primary samples is essential. Additionally, combining m6A‐seq with single‐cell and spatial RNA sequencing techniques will enable the decoding of the complexity and spatial distribution of m6A modifications within different cell subpopulations. If future advancements result in m6A‐seq technology with exceptionally high resolution and accuracy while requiring lower sample amounts, mapping m6A modifications in circulating cell‐free RNA might become feasible. This could provide m6A‐based biomarkers as noninvasive tools for early detection, diagnosis, classification, prediction, treatment selection, and monitoring therapeutic responses.

Finally, deeper molecular studies are another important direction for advancing the field of m6A. Specifically, further investigation into how m6A modifications influence posttranscriptional regulation, RNA transport, and degradation processes is needed. Moreover, studying the interactions between m6A modifications and other epigenetic marks, as well as their roles in cell signaling and disease progression, will deepen our understanding of the biological functions of m6A. By integrating high‐resolution structural biology, systems biology, and functional genomics approaches, the complex network of m6A modifications can be elucidated, offering new targets and strategies for diagnosing and treating related diseases. In summary, future research on m6A modifications should focus on improving the accessibility of technologies and further exploring the biological mechanisms, which will propel the field forward and lay the groundwork for clinical applications.

m6A‐targeted therapies have shown great promise in cancer treatment in recent years. Their advantages are primarily reflected in several aspects. First, m6A modification is a regulatory mechanism at the RNA level that can modulate cancer cell behavior by affecting gene expression, RNA stability, and translation efficiency. Targeting m6A‐related enzymes, such as METTL3 and FTO, can effectively inhibit cancer cell growth and proliferation.^299^ Additionally, the specificity of m6A modifications in certain cancer types makes it an ideal target for personalized therapies. Moreover, m6A‐targeted therapies can be combined with existing chemotherapy or immunotherapy to enhance therapeutic efficacy and reduce resistance.^310^, ^315^ However, there are limitations to m6A‐targeted therapies. First, the role of m6A modifications varies across tissues and cell types, and targeted therapy may trigger unintended side effects, particularly in normal cells. Furthermore, the dynamic and diverse nature of m6A modifications makes it challenging to precisely target and regulate m6A‐related proteins. Another limitation is that m6A‐targeted drug development is still in its early stages, and many potential drugs have not yet undergone clinical validation, with efficacy and safety still needing further research.

While several proof‐of‐concept studies highlight the potential of pharmacologically inhibiting dysregulated m6A modifications as a promising new therapeutic strategy, no inhibitors targeting m6A modifications have entered clinical trials thus far. It is understandable that developing highly selective and efficient inhibitors with a sufficient therapeutic index will take time. A better understanding of the role and mechanisms of m6A modifications in cancer will also help in selecting therapeutic targets and developing more effective treatments with minimal side effects. In addition to small‐molecule inhibitors, protein degraders offer an alternative strategy, particularly for targeting dysregulated m6A readers in cancer. Given their overlapping functions in certain cancer types, simultaneously targeting multiple oncogenic m6A modifiers is an intriguing approach to achieving optimal therapeutic outcomes. In fact, given that these m6A modifiers often contribute to tumorigenesis through different mechanisms, simultaneously targeting both oncogenic writers and erasers may be feasible and could have synergistic effects. This synergism between small‐molecule inhibitors should be validated through functional studies in models with deletions of these modifying factor genes. The recent discoveries surrounding CRISPR technologies may offer new hope for m6A therapies. If specific m6A modifications are proven to be crucial for cancer progression, using CRISPR‐based tools to manipulate these specific modifications could become a possible therapeutic approach in the future. Overall, m6A‐targeted therapy, as an emerging cancer treatment strategy, holds great potential but also faces several scientific and technical challenges that must be addressed.

## AUTHOR CONTRIBUTIONS

Xiulin Jiang, Xi Chen, Fan Zhou, and Yixiao Yuan wrote the main manuscript. Xi Chen and Fan Zhou prepared the figures and analyzed the literature in depth, and optimized the topic and structure. Lihua Li developed the conception of the topic. Xiulin Jiang and Jun Pu supervised, reviewed, and revised the written manuscript. All authors have read and approved the final manuscript.

## CONFLICT OF INTEREST STATEMENT

The authors declare no conflict of interest.

## ETHICS STATEMENT

Not applicable.

## Data Availability

Not applicable.
